# Beyond the Stomach: Exploring the Role of *Helicobacter pylori* in Pushing Precancerous Lesions to Colorectal Cancer and the Therapeutic Potential of Probiotics

**DOI:** 10.1017/erm.2026.10063

**Published:** 2026-07-02

**Authors:** Zahra Sadeghloo, Mehdi Azizmohammad Looha, Marziyeh Etesami, Georg Conrads, Sama Rezasoltani, Amir Sadeghi, Ehsan Nazemalhosseini Mojarad

**Affiliations:** 1 Basic and Molecular Epidemiology of Gastrointestinal Disorders Research Center, Research Institute for Gastroenterology and Liver Diseases, Shahid Beheshti University of Medical Sciences, Tehran, Iran; 2 Division of Oral Microbiology and Immunology, Department of Operative Dentistry, Periodontology and Preventive Dentistry, RWTH University Hospital, Aachen, Germany; 3 Gastroenterology and Liver Diseases Research Center, Research Institute for Gastroenterology and Liver Diseases, Shahid Beheshti University of Medical Sciences, Tehran, Iran

**Keywords:** colorectal cancer, gut microbiota, *Helicobacter pylori*, intracellular life, probiotics, spiral to coccoid transformation

## Abstract

Colorectal cancer (CRC), a major global health burden, is the second leading cause of cancer deaths. This review examines the link between *Helicobacter pylori* infection and CRC, highlighting its role beyond gastric pathology. Affecting over half the world’s population, *H. pylori* is associated with increased CRC risk through direct and indirect mechanisms. Direct pathways include toxins like CagA and VacA, which drive inflammation, and hypergastrinaemia, which promotes colorectal cell proliferation. Indirectly, *H. pylori* induces immune dysregulation, shifts to immune-evasive coccoid forms, survives intracellularly, releases oncogenic vesicles, disrupts autophagy, alters non-coding RNAs by dysregulating their expression profiles and contributes to gut microbiota dysbiosis. Additionally, we discuss the potential of probiotic interventions to counteract *H. pylori*’s pathogenic effects by restoring gut microbial balance, reducing inflammation and modulating immunity by regulating cytokine and T-cell profiles. Future research should translate these molecular insights into clinical applications, including evaluating whether *H. pylori* eradication reduces CRC risk in high-risk populations and assessing the preventive potential of specific probiotic strains in controlled human trials.

## Introduction

Colorectal cancer (CRC) ranks among the most prevalent tumours of the digestive system and stands as the second leading cause of cancer-related deaths worldwide (Refs 1, 2). Emerging evidence underscores the contribution of diets deficient in dietary fibre, whole grains and other protective nutrients, along with adverse lifestyle patterns, to the rising incidence and mortality associated with CRC, which remains a major public health concern (Ref. 3). The disease develops from benign or precancerous polyps in the majority of cases, which gradually transform over several years, eventually infiltrating the bowel wall, spreading to adjacent lymph nodes or metastasizing to distant organs. This prolonged progression frequently results in delayed diagnosis, whereas early detection and polypectomy of precancerous polyps can prevent CRC progression (Ref. 4). Colon polyps can be classified into low-risk and high-risk categories based on their potential to progress to CRC. This classification is essential for determining appropriate screening intervals and prevention strategies (Ref. 5). Based on their growth patterns, current histological practices categorize these lesions into two main types: serrated polyps and adenomatous polyps (APs). APs are further divided into three subtypes: villous polyps (VP), tubulovillous polyps (TVP) and tubular adenomas (TA). Similarly, serrated polyps are classified into hyperplastic polyps (HP), sessile serrated adenomas (SSA synonyms: sessile serrated lesions or polyps) and traditional serrated adenomas (TSA) (Refs 6–8). Therefore, it is crucial to identify the risk factors associated with colorectal polyps (CPs) based on their histological characteristics to enhance prevention and management strategies.

CRC risk factors can be classified into non-modifiable and modifiable risk factors. Among the non-modifiable factors are characteristics such as ethnicity, sex and age, alongside hereditary mutations and certain medical conditions like inflammatory bowel diseases (IBD), cystic fibrosis and acromegaly (Refs 9–11). Research indicates that about 5–10% of CRC instances are linked to known genetic backgrounds, while the rest occur in individuals without any notable family history or genetic predisposition (Ref. 12). In contrast, modifiable risk factors are related to lifestyle choices, such as physical inactivity, obesity and dietary habits. Additionally, smoking and alcohol use have been associated with an elevated risk of developing CRC (Refs 13–15). Moreover, the gut microbiome’s vast array of bacteria has sparked interest in how infectious agents may contribute to cancer development. This growing body of research underscores the complex interplay between genetic factors, environmental exposures and microbial influences in the pathogenesis of CRC (Ref. 16).

Recent research has indicated a possible connection between *Helicobacter pylori* infection and its role as a risk factor for adenomatous polyps and CRC (Refs 17, 18). Although *H. pylori* primarily inhabit the stomach, studies have revealed a notable correlation between *H. pylori* infection and various non-gastric diseases, such as neurodegenerative disorders, IBD, celiac disease, hepatic disorders and CRC (Refs 19–21). Epidemiological evidence underscores a significant association between *H. pylori* infection and an increased risk and aggressiveness of CRC (Refs 20, 22), with an odds ratio of 1.32 (95% CI: 1.07–1.61) for any colorectal adenoma and 1.90 (95% CI: 1.05–3.56) for advanced neoplasms and this association remains significant after adjusting for established risk factors such as smoking, alcohol consumption and body mass index (Ref. 23). Nevertheless, the specific biological mechanisms that contribute to this heightened risk are still not fully understood.

However, the interpretation of epidemiological associations between *H. pylori* infection and colorectal cancer should consider several potential confounding factors. The prevalence of *H. pylori* infection is strongly associated with socio-economic status, including sanitation conditions, educational level, healthcare access and population density, which may independently influence cancer risk and screening patterns (Ref. 24). In addition, dietary habits such as high consumption of processed or red meat, low intake of fibre, fruits and vegetables as well as lifestyle factors including smoking, alcohol consumption, obesity and physical inactivity may confound the observed relationship (Ref. 25). Furthermore, concurrent infections and alterations in the gut microbiota may co-exist with *H. pylori* infection and contribute to colorectal carcinogenesis (Ref. 26). Therefore, since most available studies are observational in nature, they cannot definitively establish causality and residual confounding cannot be fully excluded. Future longitudinal and mechanistic studies are required to clarify whether *H. pylori* play a direct causal role in colorectal tumorigenesis. While previous reviews have primarily focused on the epidemiological association between *H. pylori* and colorectal neoplasia, this article is designed as a narrative review that provides a broader and more integrated synthesis of the available evidence. This narrative review summarizes current epidemiological findings, examines the direct and indirect mechanisms through which *H. pylori* may contribute to colorectal carcinogenesis and discusses the potential role of probiotic interventions in prevention and management strategies. By integrating epidemiological, mechanistic and translational evidence, this review aims to provide a comprehensive overview of the current state of knowledge and highlight important areas for future research.

## Epidemiological evidence: connecting *H. pylori* to colorectal neoplasia

### *Helicobacter pylori* infection

*Helicobacter pylori*, a gram-negative coccoid-spiral bacterium that has co-existed with humans for over 100,000 years, is a significant player in the global landscape of gastrointestinal health (Ref. 27). *Helicobacter pylori* colonizes the gastric mucosa of over half the global population, with prevalence varying by region (Ref. 28). While colonization is common, it does not always result in disease; progression to infection and subsequent pathology depends on factors such as bacterial virulence (e.g. CagA, VacA), host immune responses and environmental conditions (Refs 28, 29). Emerging microbiome research suggests that *H. pylori* may act as a long-term gastric colonizer, potentially contributing to early-life gastric and immune development. Consequently, it may not be strictly considered an ‘infection’ unless associated with mucosal damage or symptomatic disease (Refs 30, 31). However, the transition from colonization to infection is continuous and, in this review, the term ‘infection’ is used to describe this continuous transition from colonization to infection.

Critically, beyond well-established environmental and sanitary determinants, certain modifiable lifestyle factors may influence host susceptibility to *H. pylori* infection and its persistence (Ref. 32). High-salt diets can promote *H. pylori* growth and survival and exacerbate gastric injury by promoting gastric epithelial hyperplasia, parietal cell loss and increased bacterial load (Refs 33, 34). Cigarette smoking has also been associated with altered gastric histology and a reduced humoral response to *H. pylori*, which may bacterial persistence and impair eradication (Refs 35, 36). In addition, obesity may modify host–bacterium interactions through changes in gastric hormones, including reduced ghrelin signalling and obesity-related alterations in leptin pathways, rather than directly increasing bacterial acquisition (Ref. 37).

*Helicobacter pylori*, often found nestled in the gastric mucosa, actively engages in a complex relationship with its host, contributing to chronic gastritis and serving as a precursor to more severe conditions, such as peptic ulcers and gastric cancer (GC) (Ref. 38). Recent genomic studies, including those from the *H. pylori* Genome Project, have illuminated the intricate genomic structure of this pathogen, revealing its adaptability and the mechanisms it employs to thrive in the acidic environment of the stomach (Ref. 39). Recent estimates indicate that infections account for approximately 15% of all cancer diagnoses, with *H. pylori* recognized as the most significant infectious agent associated with cancer. This bacterium is responsible for about 770,000 cancer cases globally each year, primarily due to its strong role in the development of gastric cancer (Ref. 40). *Helicobacter pylori* shows marked geographic variation, with higher prevalence in East Asia, Africa and parts of South America and lower prevalence in the United States, Oceania and Western Europe (Ref. 28). In Latin America, countries like Peru exhibit high *H. pylori* prevalence, with rates exceeding 70%, driven by factors such as sanitation challenges (Ref. 28). In Asia, China reports prevalence around 40–60% in adults, influenced by socio-economic conditions (Ref. 28). Other Eastern Mediterranean countries, such as Saudi Arabia and Egypt, show rates between 22% and 87.6%, reflecting regional hygiene issues (Ref. 41). Similarly, Iran has a notable prevalence, ranging from 54% to over 80% among adults and children, underscoring the impact of local environmental and cultural factors (Refs. 41, 42).

The persistent inflammation, mucosal injury, genetic and epigenetic modifications and altered gene expression patterns observed in individuals with *H. pylori* infection are key factors that contribute to the development of GC over time (Ref. 43). *Helicobacter pylori* have distinctive characteristics and virulence factors that enable effective infection of the host and manipulation of the immune response. The enzyme urease enables *H. pylori* to neutralize gastric acid by generating ammonia, which acts as a shield for bacterial survival while concurrently exerting a cytotoxic effect on gastric epithelial cells and disrupting mucosal tight junctions (Ref. 44). In addition, adhesion factors such as SabA (sialic acid-binding adhesin), BabA (blood group antigen-binding adhesin) and HopQ (*Helicobacter* outer membrane protein) play a pivotal role in establishing persistent gastric infection. BabA mediates bacterial attachment to Lewis B blood group antigens on gastric epithelial cells, whereas SabA binds to sialylated glycoconjugates that are up-regulated during gastric inflammation. HopQ further strengthens bacterial adherence through interaction with carcinoembryonic antigen-related cell adhesion molecules (CEACAMs) expressed on host cells (Refs. 29, 45). Furthermore, bacterial adhesion promotes host–pathogen interactions that trigger downstream intracellular signalling cascades. Specifically, outer membrane proteins and bacterial pathogen-associated molecular patterns (PAMPs) are recognized by host pattern recognition receptors (PRRs), including Toll-like receptors expressed on gastric epithelial cells (Ref. 46). This recognition event recruits downstream nuclear factor kappa B (NF-κB) and mitogen-activated protein kinase (MAPK) pathways, which orchestrate the hyper-secretion of potent pro-inflammatory cytokines and chemokines, most notably interleukin-8 (IL-8), IL-1β and tumour necrosis factor-alpha (TNF-α) (Refs 46, 47). The pathogenic potential of *H. pylori* is largely attributed to two key virulence factors: CagA (cytotoxin-associated gene A) and VacA (vacuolating cytotoxin A). The CagA oncoprotein infuses into host T cells via a type IV secretion system (T4SS) that is encoded by *cag* pathogenicity island (*cag* PAI), which negatively affects cell growth and the apoptosis process (Ref. 48). VacA, when internalized by host T cells, induces various adverse effects, such as cell vacuolation, which enhances the persistence of infection, along with mitochondrial dysfunction, stress and, ultimately, apoptosis (Ref. 49). This continuous molecular and cellular crosstalk drives a sustained influx of neutrophils, macrophages and lymphocytes into the lamina propria. The persistent activation of these immune cells generates chronic oxidative stress through the excessive release of reactive oxygen and nitrogen species (ROS/RNS), leading to cumulative mucosal damage and maintaining the chronic active gastritis that serves as the foundation for downstream neoplasia (Ref. 50). Evidence increasingly suggests that *H. pylori* infection may be linked to CRC (Refs 29, 51, 52). Although a single definitive pathway has not been universally established, this oncogenic association is postulated to rely on several inter-related mechanisms. Rather than driving carcinogenesis through a localized event, this correlation appears to involve an extended physiological network that operates beyond the stomach, where the bacterium first interacts with the host. In this context, it is crucial to distinguish the conceptual and pathophysiological boundaries between *H. pylori*-induced GC and its hypothesized role CRC. In gastric carcinogenesis, the pathogen’s oncogenic role is locally restricted to the gastric niche, occurring within a highly acidic environment and directly driving the well-established, sequential Correa’s cascade (Ref. 53). Conversely, the biological landscape of CRC is fundamentally distinct, presenting a more complex and expanded paradigm; as discussed in detail later in this review, the link to colorectal neoplasia involves a sophisticated combination of both indirect systemic alterations and direct distal actions. Consequently, moving past a simple blanket assumption to closely evaluate how these broad pathways are structured in current literature is essential to appreciate their full clinical significance. This review aims to explore the complex relationship between *H. pylori* infection and CRC. To illuminate this gastrointestinal field of research, we will first examine in detail the association between *H. pylori* infection and the development of colorectal polyps and cancer. Next, we will discuss the proposed biological pathways, including both direct and indirect mechanisms, that have been hypothesized to link *H. pylori* infection with CRC development. Finally, we will investigate the role of probiotics in modulating *H. pylori* pathogenesis and their potential to improve CRC management.

### Association between *H. pylori* infection and colorectal neoplasia

Table 1 summarizes the available evidence on the association between *H. pylori* infection and colorectal neoplasia, including crude and adjusted odds ratios extracted from the individual studies included in this review. The evidence was organized according to major colorectal outcome categories, including neoplasms, advanced neoplasms, CRC, overall colorectal polyps, adenoma polyps, advanced adenoma polyps, conventional adenoma polyps, hyperplastic polyps and SSAs. These categories reflect the polyp-to-CRC progression model and the histological classifications commonly used in the literature and recent meta-analyses. Although some overlap exists across sub-groups, this structure allows a clearer synthesis of the available evidence. Associations according to polyp number and size were also reviewed. Because the covariates included in adjusted models varied across publications, adjusted odds ratios are presented descriptively and should be interpreted within the context of each individual study rather than compared directly across studies. These findings are summarized next according to outcome category and, where available, crude and adjusted estimates.

**Table 1.** Reported crude and adjusted odds ratios for the association between *Helicobacter pylori* infection and colorectal neoplasia across individual studies Table 1. long description.

Study design	Outcome category	Sample size		Crude			Adjusted			Ref
		Cases	Control	OR	Lower	Upper	OR	Lower	Upper	
Case–control	Neoplasms (adenoma polyp + CRC)	127	470	1.94	1.28	2.95	1.90	1.23	2.93	(Ref. 54)
Case–control	Neoplasms (adenoma polyp + CRC)	481	18				1.66	1.27	2.05	(Ref. 55)
Cross-sectional	Neoplasms (adenoma polyp + CRC)	112	161	4.07	2.26	7.35				(Ref. 52)
Cross-sectional	Neoplasms (adenoma polyp + CRC)	2,497	3,854	1.33	1.19	1.48	1.22	1.09	1.36	(Ref. 56)
Case–control	Advanced neoplasms (advanced adenoma + CRC)	30	470	2.40	0.64	8.97	2.39	0.69	9.21	(Ref. 54)
Cross-sectional	Advanced neoplasms (advanced adenoma + CRC)	224	5,713				1.90	1.05	3.56	(Ref. 22)
Cross-sectional	Advanced neoplasms (advanced adenoma + CRC)	46	1,798	1.11	0.03	2.26				(Ref. 57)
Cross-sectional	Advanced neoplasms (advanced adenoma + CRC)	77	161	9.75	4.31	21.20				(Ref. 52)
Cross-sectional	Advanced neoplasms (advanced adenoma + CRC)	316	3,854	1.49	1.17	1.91	1.34	1.04	1.72	(Ref. 56)
Retrospective	CRC	82,420	47,632,330	3.92	3.52	4.63	1.89	1.69	2.10	(Ref. 58)
Case–control	CRC	1,682	1,606	1.09	0.99	1.20				(Ref. 51)
Case–control	CRC	9	470	1.57	0.39	6.35	1.54	0.36	6.51	(Ref. 54)
Retrospective	CRC	27	95	1.46	0.38	5.54				(Ref. 59)
Prospective cohort	CRC	485	485	1.41	1.06	1.89	1.36	1.00	1.85	(Ref. 60)
Meta-analysis	CRC	384	467				1.41	1.06	1.87	(Ref. 17)
Case–control	CRC	67	92				10.60	2.70	41.30	(Ref. 61)
Prospective	CRC	159	158				0.90	0.50	1.50	(Ref. 62)
Case–control	CRC	154	188				1.80	1.28	2.32	(Ref. 55)
Case–control	CRC	56	58	8.13	1.40	46.99				(Ref. 63)
Case–control	CRC	189	179	1.10	0.70	1.80				(Ref. 64)
Case–control	CRC	329	774				1.17	0.91	1.50	(Ref. 65)
Case–control	CRC	188	370	1.03	0.59	1.77				(Ref. 66)
Case–control	CRC	946	65,502	2.35	1.98	2.80				(Ref. 67)
Retrospective	CRC	304	2,362				3.05	2.33	3.99	(Ref. 68)
Cross-sectional	CRC			1.12	0.98	1.27				(Ref. 69)
–	CRC	1,712	1,669				1.30	1.14	1.50	(Ref. 70)
Cross-sectional	CRC	44	161	7.98	3.16	20.16				(Ref. 52)
Retrospective cross-sectional	CRC	74	3,707				1.67	0.98	2.80	(Ref. 71)
Retrospective cohort	CRC	1,044	7,122				1.34	1.17	1.53	(Ref. 72)
Case–control	CRC	392	774	1.17	0.91	1.50				(Ref. 65)
Case–control	CRC	41	41	0.74	0.28	1.96				(Ref. 73)
Case–control	CRC	95	108	2.23	1.17	4.27				(Ref. 74)
–	CRC	135					1.56	1.06	2.30	(Ref. 75)
Case–control	Overall colorectal polyps	57	179	1.30	0.70	2.50				(Ref. 76)
Retrospective	Overall colorectal polyps	3,872	2,362				2.19	1.96	2.44	(Ref. 68)
Case–control	Overall colorectal polyps	1,051	3,221				1.13	1.10	1.52	(Ref. 77)
Cross-sectional	Overall colorectal polyps			1.25	1.12	1.40				(Ref. 78)
Retrospective cohort	Overall colorectal polyps	456	800	1.30	1.00	1.60	1.50	1.20	1.90	(Ref. 79)
Cross-sectional	Overall colorectal polyps	77	226	2.26	1.32	3.86				(Ref. 80)
Retrospective	Overall colorectal polyps	330	263	5.23	3.60	7.70				(Ref. 81)
–	Overall colorectal polyps	3,483					1.17	1.04	1.32	(Ref. 75)
Case–control	Adenoma polyps	11	470	1.96	1.27	3.01	1.93	1.24	3.01	(Ref. 54)
Cross-sectional	Adenoma polyps	3,203	5,713				1.32	1.07	1.61	(Ref. 22)
Case–control	Adenoma polyps	1,000	1,500				1.17	1.13	4.27	(Ref. 82)
Case–control	Adenoma polyps	327	188				1.60	1.18	2.02	(Ref. 55)
Cross-sectional	Adenoma polyps	319	665	1.44	1.20	1.73	1.42	1.18	1.71	(Ref. 83)
Case–control	Adenoma polyps	94	94	1.98	0.82	3.15				(Ref. 84)
Case–control	Adenoma polyps	66,221	65,502	1.52	1.46	1.57				(Ref. 67)
Case–control	Adenoma polyps	1,245	3,221				1.28	1.11	1.47	(Ref. 77)
Retrospective	Adenoma polyps	297	1,058	1.54	1.04	1.75				(Ref. 85)
Retrospective	Adenoma polyps	1,012	3,983	1.19	1.04	1.37				(Ref. 86)
Cross-sectional	Adenoma polyps	1,195	180	2.86	1.98	4.15	3.24	2.18	4.80	(Ref. 87)
Retrospective	Adenoma polyps	55	120				1.04	0.67	1.60	(Ref. 88)
Cross-sectional	Adenoma polyps	506	1,689	1.35	1.10	1.66	1.36	1.10	1.68	(Ref. 89)
Retrospective cohort	Adenoma polyps	300	956	1.70	1.30	2.20	1.50	1.10	2.00	(Ref. 79)
Retrospective	Adenoma polyps	286	263	3.20	1.60	6.40				(Ref. 81)
Retrospective cross-sectional	Adenoma polyps	1,516	3,707				1.22	1.08	1.38	(Ref. 71)
Retrospective case–control	Adenoma polyps	216	727	1.43	1.04	1.97	1.55	1.13	2.12	(Ref. 90)
Case–control	Adenoma polyps	239	239	2.26	1.44	3.55	2.52	1.57	4.05	(Ref. 91)
Prospective case–control	Adenoma polyps	78	78	3.20	1.40	7.50				(Ref. 92)
Cross-sectional	Adenoma polyps	1,923	7,388	1.37	1.23	1.52				(Ref. 93)
Case–control	Adenoma polyps	41	41	1.00	0.37	2.70				(Ref. 73)
Case–control	Advanced adenoma polyps	9,949	65,502	1.80	1.69	1.92				(Ref. 67)
Case–control	Advanced adenoma polyps	118	3,221				1.84	1.25	2.70	(Ref. 77)
Retrospective	Advanced adenoma polyps	143	3,983	1.27	0.09	1.77				(Ref. 86)
Retrospective	Advanced adenoma polyps	28	120				0.59	0.32	1.12	(Ref. 88)
Cross-sectional	Advanced adenoma polyps	103	1,689	2.19	1.40	3.42	1.48	1.41	2.21	(Ref. 89)
Retrospective cohort	Conventional adenoma polyps	20,363	7,122				1.11	1.05	1.17	(Ref. 72)
Case–control	Hyperplastic polyp	23,601	65,502	1.24	1.18	1.30				(Ref. 67)
Cross-sectional	Hyperplastic polyp	42	226	0.14	0.03	0.66				(Ref. 80)
Retrospective cohort	Hyperplastic polyp	43,329	138,893	1.27	1.22	1.32	1.23	1.18	1.28	(Ref. 94)
Prospective	Hyperplastic polyp	29	244	2.26	1.23	5.74				(Ref. 95)
Retrospective	Single polyp	1,766	2,362				1.98	1.74	2.25	(Ref. 68)
Retrospective	Single polyp	319	1,058	1.33	1.03	1.71				(Ref. 85)
Observational	Single polyp	431	829	1.60	1.32	2.11				(Ref. 96)
Retrospective	Single polyp	1,030	3,983	1.08	0.94	1.24				(Ref. 86)
Retrospective	Two or more polyps	2,106	2,362				2.41	2.12	2.74	(Ref. 68)
Retrospective	Two or more polyps	264	1,058	1.31	1.00	1.71				(Ref. 85)
Observational	Two or more polyps	1,362	829	2.10	1.74	2.58				(Ref. 96)
Retrospective	Two or more polyps	1,005	3,983	1.15	1.00	1.32				(Ref. 86)
Case–control	Polyps ≥10 mm	933	2,362				2.33	1.98	2.74	(Ref. 68)
Retrospective	Polyps ≥10 mm	42	1,058	0.94	0.51	1.75				(Ref. 85)
Retrospective	Polyps ≥10 mm	147	3,983	1.33	0.96	1.86				(Ref. 86)
Retrospective	Polyps <10 mm	2,939	2,362				2.15	1.92	2.41	(Ref. 68)
Retrospective	Polyps <10 mm	541	1,058	1.35	1.10	1.67				(Ref. 85)
Retrospective	Polyps <10 mm	1,888	3,983	1.10	0.99	1.23				(Ref. 86)

*Note*: Adjusted odds ratios were extracted as reported in the original publications. The covariates included in the multivariable models differed across studies and were not standardized. Therefore, adjusted estimates should be interpreted within the context of each individual study and should not be directly compared across publications as equivalent effect estimates.

#### Neoplasms (adenoma polyp + CRC)

Several studies evaluated the association between *H. pylori* and the broad category of colorectal neoplasms, which includes adenoma polyps and CRC (Refs 52, 54–56). Crude ORs reported by Nam et al. (Ref. 54) (OR = 1.94, 95% CI: 1.28–2.95), Shmuely et al. (Ref. 52) (OR = 4.07, 95% CI: 2.26–7.35) and Lee et al. (Ref. 56) (OR = 1.33, 95% CI: 1.19–1.48) indicate an increased risk of colorectal neoplasia among individuals with *H. pylori* infection. Several studies also reported adjusted estimates above 1, including Nam et al. (Ref. 54) (OR = 1.90, 95% CI: 1.23–2.93), Fujimori et al. (Ref. 55) (OR = 1.66, 95% CI: 1.27–2.05) and Lee et al. (Ref. 56) (OR = 1.22, 95% CI: 1.09–1.36). However, these adjusted estimates were derived from study-specific models with different covariate sets and should therefore be interpreted as within-study findings rather than directly comparable measures across publications.

However, the magnitude of association varies considerably across studies, ranging from modest associations to stronger estimates, suggesting underlying heterogeneity (Refs 52, 54–56). This variability may reflect differences in study design (case–control vs. cohort), geographic distribution (e.g. East Asia vs. Western populations) and baseline prevalence of both *H. pylori* infection and colorectal neoplasia, which can influence effect estimates and limit direct comparability.

#### Advanced neoplasms (advanced adenoma + CRC)

Advanced neoplasms, including high-risk adenomas and CRC, showed mixed findings across studies (Refs 22, 52, 54, 56, 57). Nam et al. (Ref. 54) reported an OR of 2.40 (95% CI: 0.64–8.97) and Park et al. (Ref. 57) reported an OR of 1.10 (95% CI: 0.02–2.26); however, both confidence intervals were wide and crossed 1, indicating substantial statistical uncertainty and no clear evidence of increased risk in these studies. In contrast, Shmuely et al. (Ref. 52) reported a markedly increased risk of advanced neoplasms among individuals with *H. pylori* infection (OR = 9.75, 95% CI: 4.31–21.20). Adjusted estimates were also reported by Kim et al. (Ref. 22) (OR = 1.90, 95% CI: 1.05–3.56), Lee et al. (Ref. 56) (OR = 1.34, 95% CI: 1.04–1.72) and Nam et al. (Ref. 54) (OR = 2.39, 95% CI: 0.69–9.21). Because these models did not account for an identical set of covariates, they provide study-specific evidence rather than directly comparable adjusted contrasts.

Overall, the evidence for advanced neoplasms should be interpreted cautiously because some estimates were imprecise, with wide confidence intervals that crossed 1, while stronger positive findings were reported only in selected studies (Refs 22, 52, 54, 56, 57).

#### Colorectal cancer

Several studies have assessed the association between *H. pylori* infection and CRC, with heterogeneous findings across populations and study designs (Refs 51, 58, 60). Crude ORs reported by Jones et al. (Ref. 63) (OR = 8.13, 95% CI: 1.40–46.99), Shmuely et al. (Ref. 52) (OR = 7.98, 95% CI: 3.16–20.16) and Sonnenberg et al. (Ref. 67) (OR = 2.35, 95% CI: 1.98–2.80) indicate an increased risk of CRC among individuals with *H. pylori* infection. Adjusted analyses show similar results, with Wang et al. (Ref. 68) (OR = 3.05, 95% CI: 2.33–3.99), Shmuely et al. (Ref. 61) (OR = 10.60, 95% CI: 2.70–41.30) and Pan et al. (Ref. 72) (OR = 1.34, 95% CI: 1.17–1.53) indicating an increased risk of CRC in the corresponding study populations. Several studies reported ORs above 1 for the association between *H. pylori* infection and CRC; however, the magnitude of association varied substantially across studies, and the adjusted estimates were based on non-uniform covariate sets (Refs 58, 60, 63, 67, 68).

However, this apparent robustness must be interpreted cautiously, as effect sizes vary markedly across studies, ranging from null or weak associations to very strong associations, indicating substantial heterogeneity (Refs 54, 58, 66, 67). Such discrepancies may arise from differences in exposure assessment (e.g. serology reflecting past exposure vs. urea breath test reflecting active infection), outcome definition and population risk profiles.

#### Overall colorectal polyps

Crude ORs for overall colorectal polyps indicate an increased risk of polyp formation among individuals with *H. pylori* infection, as reported by Ciftel et al. (Ref. 81) (OR = 5.23, 95% CI: 3.60–7.70), Tongtawee et al. (Ref. 80) (OR = 2.26, 95% CI: 1.32–3.86) and Brim et al. (Ref. 79) (OR = 1.30, 95% CI: 1.00–1.60). Adjusted analyses confirm these findings, with Wang et al. (Ref. 68) (OR = 2.19, 95% CI: 1.96–2.44) and Brim et al. (Ref. 79) (OR = 1.50, 95% CI: 1.20–1.90) showing statistical significance. These findings support the hypothesis that *H. pylori* infection may contribute to early-stage colorectal neoplasia by promoting polyp formation. Nevertheless, these estimates arose from different analytic models and should be interpreted within the methodological context of each study.

#### Adenoma polyps

The relationship between *H. pylori* and adenoma polyps was also evident. Crude ORs reported by Nam et al. (Ref. 54) (OR = 1.96, 95% CI: 1.27–3.01), Chen et al. (Ref. 86)(OR = 2.86, 95% CI: 1.97–4.15) and Inoue et al. (Ref. 91) (OR = 2.26, 95% CI: 1.44–3.55) indicate an increased risk of adenoma polyps among individuals with *H. pylori* infection. Adjusted analyses reinforce this, with Chen et al. (Ref. 86) (OR = 3.23, 95% CI: 2.18–4.79) and Inoue et al. (Ref. 91) (OR = 2.52, 95% CI: 1.57–4.05) showing strong correlations. The data indicate that *H. pylori* infection is a significant risk factor for adenoma polyps, a crucial precursor in colorectal carcinogenesis. Because the multivariable models differed between studies, these estimates should be viewed as separate study-level findings rather than directly comparable measures.

However, variability in effect sizes and study populations suggests that these associations may not be uniform across different demographic or geographic contexts.

#### Advanced adenoma polyps

Crude ORs for advanced adenomas varied across studies. While Hong et al. (Ref. 89) (OR = 2.19, 95% CI: 1.40–3.42) reported an increased risk of advanced adenomas among individuals with *H. pylori* infection, other studies reported weaker or non-significant findings, such as Chen et al. (Ref. 86) (OR = 1.26, 95% CI: 0.09–1.77). This inconsistency highlights the potential influence of methodological heterogeneity, including differences in polyp classification criteria and diagnostic protocols, which may affect comparability across studies. Similarly, Sonnenberg et al. (Ref. 67) (OR = 1.80, 95% CI: 1.69–1.92) reported an increased risk of advanced adenomas, whereas Bae et al. (Ref. 88) (OR = 0.59, 95% CI: 0.31–1.12) showed no significant relationship. Study-specific adjusted estimates also showed mixed findings. Nam et al. (Ref. 77) (OR = 1.84, 95% CI: 1.25–2.70) and Hong et al. (Ref. 89) (OR = 1.48, 95% CI: 1.41–2.21) reported increased risk estimates in their respective adjusted models. However, these adjusted estimates were derived from study-specific multivariable analyses with different covariate sets and should not be directly contrasted across publications. In contrast, Bae et al. (Ref. 88) reported an adjusted OR of 0.59 (95% CI: 0.31–1.12), which did not indicate a statistically significant increased risk of advanced adenomas. Overall, the evidence for advanced adenoma polyps is mixed; some studies reported increased risk estimates, whereas others showed non-significant findings with confidence intervals crossing 1.

#### Conventional adenoma polyps

Pan et al. (Ref. 72) reported a modestly increased risk of conventional adenoma polyps among individuals with *H. pylori* infection (OR = 1.11, 95% CI: 1.05–1.17). However, the available evidence for this outcome is limited and the observed effect size was small. Therefore, additional well-designed prospective studies are needed to determine whether this association is reproducible across different populations and whether it remains significant after standardized adjustment for major confounders, including age, sex, body mass index, smoking, alcohol use, dietary factors, socio-economic status, medication use and colorectal screening history. Future studies should also distinguish active *H. pylori* infection from past exposure and examine whether bacterial virulence factors, such as CagA status, modify the risk of conventional adenoma formation. These studies are needed because conventional adenomas are important precursors of CRC, but the current evidence is insufficient to determine whether *H. pylori* has an independent role in their development or whether the observed association reflects residual confounding, exposure misclassification or differences in study design.

#### Sessile serrated adenomas/ polyps

The role of *H. pylori* in SSAs/polyps remains unclear because the available studies reported inconsistent results. Dent et al. (Ref. 94) reported a crude OR close to the null value (OR = 1.02, 95% CI: 0.96–1.10), suggesting little or no association between *H. pylori* infection and SSAs. In contrast, Pan et al. (Ref. 72) reported an adjusted estimate indicating a modest but statistically significant increased risk of SSAs (OR = 1.41, 95% CI: 1.22–1.63). These contradictory findings may be explained by differences in study design, population characteristics, outcome definition, sample size and statistical adjustment. In particular, Dent et al. reported a crude estimate, whereas Pan et al. reported an adjusted estimate derived from a retrospective cohort model. Therefore, these results should not be interpreted as directly comparable. Differences in colorectal screening practices, histopathological classification of serrated lesions and the method used to define *H. pylori* exposure may also have contributed to the inconsistent findings. Therefore, the evidence for SSAs remains limited and further studies using standardized definitions of serrated lesions and uniform adjustment for confounders are needed.

#### Hyperplastic polyps

The relationship between *H. pylori* infection and hyperplastic polyps is inconsistent across studies. Sonnenberg et al. (Ref. 67) reported a modest increased risk of hyperplastic polyps (OR = 1.24, 95% CI: 1.18–1.30) and Selgrad et al. (Ref. 95) also reported an increased risk (OR = 2.26, 95% CI: 1.23–5.74). Similarly, Dent et al. (Ref. 94) reported a modest increased risk in an adjusted analysis (OR = 1.23, 95% CI: 1.18–1.28). In contrast, Tongtawee et al. (Ref. 80) reported an inverse association (OR = 0.14, 95% CI: 0.03–0.66). These conflicting findings may reflect important differences between the studies, including geographic setting, study design, sample size, patient selection, indication for colonoscopy, background prevalence of *H. pylori* infection and diagnostic criteria for hyperplastic polyps. The studies also differed in whether they reported crude or adjusted estimates, and the covariates included in adjusted models were not uniform. In addition, variation in *H. pylori* detection methods may have contributed to exposure misclassification, particularly when tests reflected previous exposure rather than active infection. Therefore, the evidence does not support a consistent conclusion regarding hyperplastic polyps, and future studies should use standardized histological definitions, clearly defined *H. pylori* exposure assessment and comparable multivariable adjustment.

#### Role of number and size of polyps

Data on polyp number and size were extracted from the individual studies summarized in Table 1, particularly Wang et al. (Ref. 68), Yan et al. (Ref. 58), Basmaci et al. (Ref. 96) and Chen et al. (Ref. 86). No new pooled or secondary statistical analysis was performed for these variables in this review. Instead, we descriptively summarized the crude and adjusted odds ratios reported in the original publications. Polyp number was categorized as single polyp or two or more polyps, while polyp size was categorized as <10 mm or ≥10 mm, according to the definitions used in the included studies (Refs 46, 52, 58, 63). Because the original studies differed in study design, population characteristics, *H. pylori* assessment and covariate adjustment, these estimates should be interpreted as study-level findings rather than directly comparable pooled estimates.

#### Single polyp

The association between *H. pylori* infection and single polyp formation was evaluated in several studies, with mixed findings. Crude estimates from two studies showed odds ratios above 1, suggesting a possible increased occurrence of single polyps among individuals with *H. pylori* infection (Refs 85, 96). In contrast, another study reported no statistically significant association (OR = 1.08, 95% CI: 0.94–1.24) (Ref. 86). Wang et al. reported an adjusted odds ratio of 1.98 (95% CI: 1.74–2.25) (Ref. 68); however, this estimate was extracted directly from the original publication and was not re-analysed or re-adjusted in this review. Therefore, the evidence regarding single polyp formation should be interpreted cautiously as study-level findings rather than as a definitive or pooled association.

#### Two or more polyps

A stronger association was observed when multiple polyps were considered. Crude ORs from Basmac et al. (Ref. 96) (OR = 2.10, 95% CI: 1.74–2.58) and Chen et al. (Ref. 86) (OR = 1.15, 95% CI: 1.00–1.32) demonstrate a higher risk of polyp multiplicity in *H. pylori*-infected individuals. Adjusted ORs from Wang et al. (Ref. 68) (OR = 2.41, 95% CI: 2.12–2.74) further support this finding.

#### Polyps ≥ 10 mm

Crude ORs for polyps ≥10 mm showed non-significant results. Yan et al. (Ref. 85) (OR = 0.94, 95% CI: 0.50–1.75) and Chen et al. (Ref. 86) (OR = 1.33, 95% CI: 0.95–1.85) found no significant association. Adjusted analyses from Wang et al. (Ref. 68) (OR = 2.33, 95% CI: 1.98–2.74) reported an increased risk of polyps ≥10 mm among individuals with *H. pylori* infection.

#### Polyps < 10 mm

For smaller polyps, crude ORs showed mixed results. Yan et al. (Ref. 85) (OR = 1.35, 95% CI: 1.09–1.66) reported an increased risk of small polyp formation among individuals with *H. pylori* infection, while Chen et al. (Ref. 86) (OR = 1.10, 95% CI: 0.98–1.22) did not find a statistically significant increase. Wang et al. (Ref. 68) (OR = 2.15, 95% CI: 1.92–2.41) reported an increased risk of polyps <10 mm among individuals with *H. pylori* infection.

The increased risk estimates reported in several crude and adjusted analyses suggest that *H. pylori* infection may be associated with a higher occurrence of colorectal neoplastic outcomes beyond its well-established impact on gastric pathology; however, these findings reflect epidemiological associations rather than direct evidence that *H. pylori* play a causal role in colorectal carcinogenesis. Several studies reported increased odds ratios for the association between *H. pylori* infection and advanced colorectal neoplasms, particularly high-risk adenomas and CRC; however, the strength of this association varied across studies and should be interpreted cautiously because of heterogeneity in study design, exposure assessment, outcome definition and confounder adjustment. However, while some sub-categories of colorectal polyps, such as SSAs and HPs, exhibited moderate or inconsistent associations with *H. pylori* infection, the overall trend points to an increased risk of neoplastic progression. Importantly, the magnitude and direction of these associations vary substantially across studies, reflecting significant heterogeneity in study populations, including differences in ethnicity, geographic region, baseline risk and healthcare access, all of which may influence both exposure prevalence and CRC detection. The differences observed in adjusted odds ratios across studies further emphasize the need for standardized methodologies in future research to control for these confounders effectively. Moreover, although many studies report adjusted estimates, the range and quality of confounder adjustment are inconsistent and key factors such as diet, antibiotic exposure, screening practices and gut microbiota composition are not uniformly accounted for, leaving the possibility of residual confounding. Variability in ORs may stem from methodological differences, such as diverse *H. pylori* detection methods (e.g. serology vs. urea breath test), study designs (case–control vs. cohort) or population-specific factors like antibiotic resistance and dietary habits, necessitating standardized protocols to ensure comparable results (Ref. 97). In particular, the use of serological assays in some studies may reflect past exposure rather than active infection, potentially leading to exposure misclassification and biasing the observed associations. Further investigations, particularly prospective longitudinal studies, are needed to establish a causal relationship and elucidate the precise biological mechanisms underlying *H. pylori*-induced colorectal carcinogenesis. Given the high *H. pylori* prevalence in Middle Eastern and developing countries, region-specific studies are critical to assess whether unique risk factors, like dietary patterns or socio-economic conditions, modulate the *H. pylori*-CRC association in these populations (Refs 41, 42). Additionally, studies examining the impact of *H. pylori* eradication on CRC risk reduction could provide valuable insights into potential preventive strategies. Emerging evidence suggests *H. pylori* eradication may reduce CRC risk, as a Taiwanese study found a threefold higher colorectal adenoma incidence in patients with persistent infection compared to those with successful eradication, though data remain limited (Ref. 98). At the same time, the presence of both strong positive associations and null findings across the literature indicates that this relationship is not entirely consistent and may be context dependent or influenced by study design limitations and unmeasured biases. Understanding these interactions will be crucial in developing targeted interventions for individuals at high risk of CRC, particularly those with a history of *H. pylori* infection. Ultimately, these findings support the growing recognition of *H. pylori* infection as a possible factor associated with colorectal neoplasia, while also highlighting that current epidemiological evidence remains insufficient to establish a definitive causal relationship.

Despite the consistent associations observed across multiple studies, these findings should be interpreted with caution due to the inherent limitations of observational epidemiological designs. Most of the included studies are case–control or cohort studies, which are susceptible to residual confounding and cannot establish causality. In addition, the covariates included in adjusted analyses differed across studies, limiting the direct comparability of multivariable estimates summarized in Table 1. Several potential confounders may influence the observed relationship between *H. pylori* infection and colorectal neoplasia, including dietary patterns (e.g. high red meat intake, low fibre consumption), socio-economic status (affecting sanitation, healthcare access and screening practices), lifestyle factors such as smoking, alcohol use and obesity, as well as the presence of concurrent infections and underlying alterations in gut microbiota. Furthermore, heterogeneity in diagnostic methods for *H. pylori* detection and variability in study populations may contribute to inconsistent findings across studies. Therefore, while the epidemiological evidence suggests a possible association, a direct causal relationship remains unproven and requires confirmation through well-designed longitudinal and mechanistic studies.

### Controversy in the literature: A balanced view

Although prior sections have presented substantial evidence supporting an association between *H. pylori* infection and CRC or colorectal polyps, some studies report no significant link, highlighting the complexity of this relationship. Notably, Luo et al. (Ref. 99) conducted a bidirectional Mendelian randomization study and found no causal association between *H. pylori* infection and CRC (OR = 1.12, 95% CI: 0.98–1.27, *P* = 0.08). Similarly, Blase et al. (Ref. 65) reported no association between pre-diagnostic *H. pylori* antibodies and CRC risk in an elderly Caucasian population (OR = 1.17, 95% CI: 0.91–1.50). Additionally, Guo et al. (Ref. 100) found no statistical association with CRC in East Asian populations (OR = 1.08, 95% CI: 0.89–1.68), though an association with colorectal adenomas was noted. Boyuk et al. (Ref. 101) also reported no significant relationship between *H. pylori* infection and colorectal neoplasms, including CRC and polyps (*P* > 0.05). These findings may be limited by factors such as reliance on genetic or serological methods that may not fully capture active infections or by population-specific characteristics that reduce generalizability. Inconsistencies in the literature likely arise from variations in study design, population demographics, detection methods for *H. pylori* and colorectal outcomes, and unaccounted confounders like diet or lifestyle. Despite these dissenting studies, the broader scientific consensus, supported by extensive epidemiological and mechanistic evidence, suggests that *H. pylori* infection contributes to an increased risk of CRC and colorectal polyps, emphasizing the need for further research to resolve these discrepancies and confirm the association.

These discrepancies further highlight the limitations of observational evidence and the potential impact of unmeasured or residual confounding. Collectively, current data highlight the need for cautious interpretation of epidemiological associations in the absence of robust causal inference.

## Proposed mechanistic pathways linking *H. pylori* infection to colorectal carcinogenesis

**Figure 1.**
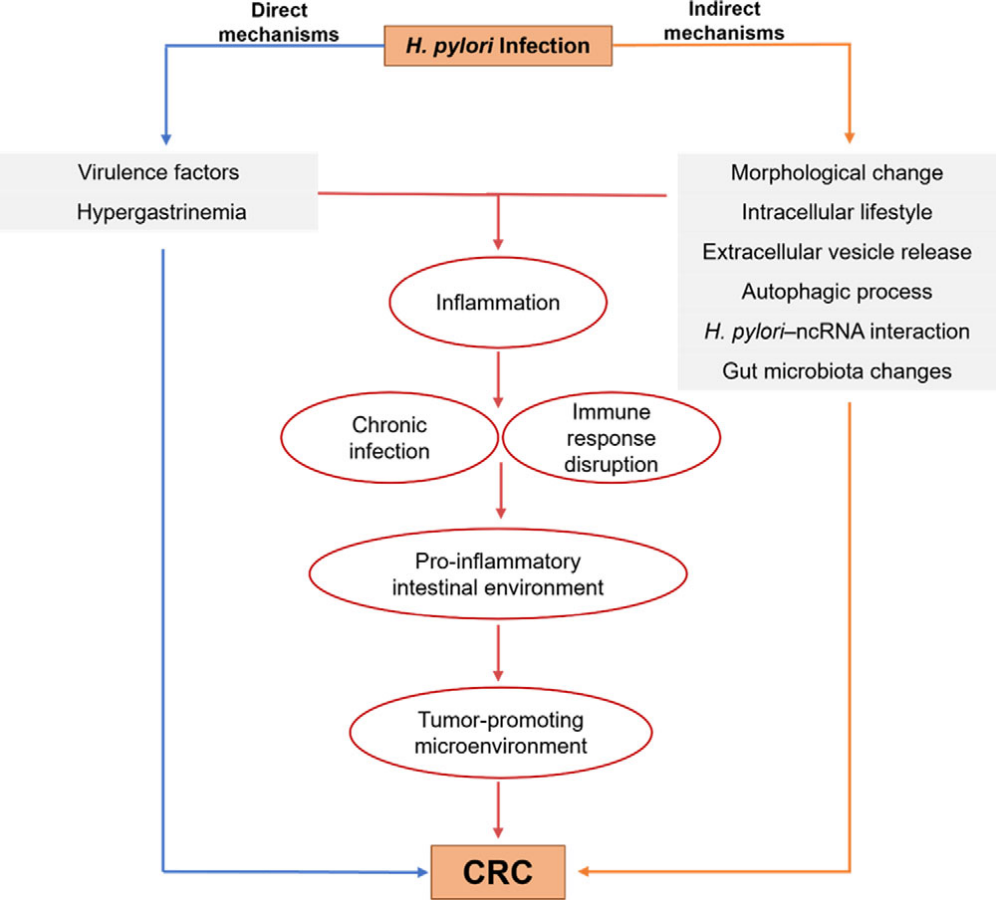
Proposed flowchart illustrating the relationship between *Helicobacter pylori* infection and CRC. The blue pathway represents direct mechanisms and the orange pathway represents indirect mechanisms through which *H. pylori* may contribute to colorectal carcinogenesis. Despite their different origins, these pathways converge at intestinal inflammation, which promotes chronic infection, immune response disruption and the establishment of a pro‑inflammatory, tumour‑promoting intestinal microenvironment that ultimately facilitates CRC development. Figure 1. long description.

**Figure 2.**
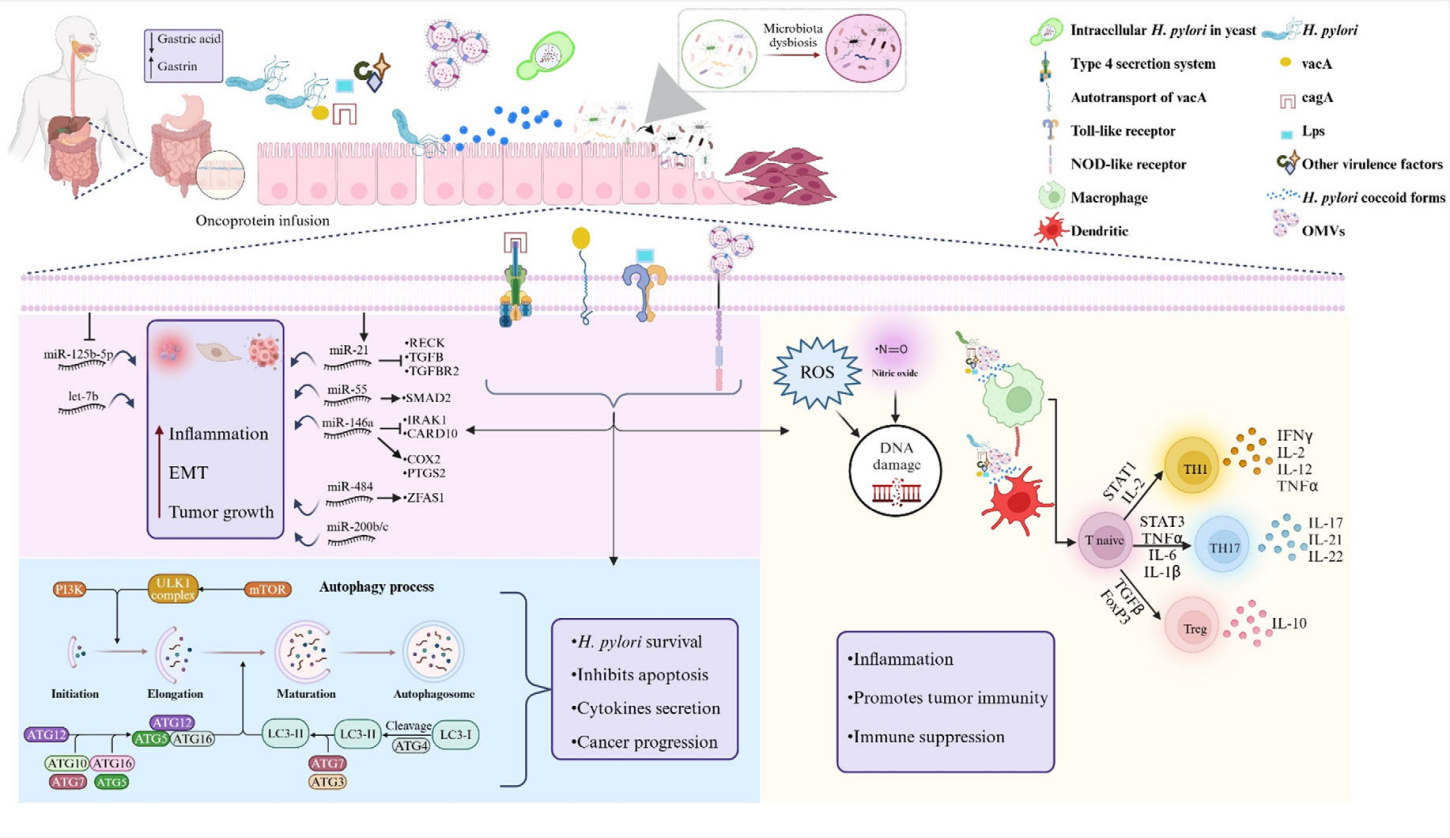
An overview of *Helicobacter pylori*’s mechanisms contributing to CRC pathogenesis. *Helicobacter pylori* contributes to CRC development through multiple mechanisms, including genotoxic effects and hypergastrinaemia induced by the bacterium’s direct presence and secreted toxins. Additionally, *H. pylori* modulates immune responses, creating an inflammatory microenvironment and promoting tumorigenesis in colorectal tissue. These effects are mediated through morphological alterations, intracellular survival, release of OMVs, modulation of host autophagy, impacts on non-coding RNAs and changes in the intestinal microbiota. Created by biorender.com. Figure 2. long description.

The mechanisms discussed in this section are proposed pathways derived from experimental, observational and pre-clinical evidence. While these findings provide biologically plausible links between *H. pylori* infection and colorectal carcinogenesis, most remain hypothetical and require further validation in human studies (Figures 1 and 2).

### Direct mechanisms

Immunohistochemical analyses have detected *H. pylori* in TA, TVP, VP polyps and adenocarcinomas (Refs 63, 102). Additionally, a notable association exists between *H. pylori* gastritis and various types of polyps – including HP, APs, advanced adenomas and high-grade dysplasia – which may indicate a potential role for this pathogen in CRC (Refs 67, 103). The detection of *H. pylori* DNA and antigens in colorectal tissues and cancers prompts a deeper exploration into its potential direct role in the mechanisms underlying tumorigenesis (Ref. 104). Two main routes have been proposed for the direct mechanisms by which *H. pylori* contribute to CRC: (1) the direct secretion of bacterial toxins that affect colorectal tissue and (2) systemic alterations, such as hypergastrinaemia, that create a favourable environment for carcinogenesis in the gastrointestinal tract.

#### Secreted toxins of H. pylori

The presence of *H. pylori* or its active molecules, such as secreted toxins, within the colorectal tissue signifies a direct effect. The possibility that *H. pylori* or its components can migrate through the colon is substantiated by multiple lines of evidence. Grahn et al. (Ref. 105) reported that *H. pylori* was found in 22%–27% of analysed CPs or cancers. In a separate case–control study comparing cancer patients to healthy controls, researchers found that *H. pylori* were present in the tumour tissues of 19 out of 118 individuals diagnosed with cancer. In contrast, only 1 out of 58 healthy individuals showed evidence of *H. pylori* in their colon tissue (Ref. 63).

CagA: An animal study employing mouse models of intestinal inflammation has provided evidence that certain pathogenic factors of *H. pylori* can influence areas beyond the stomach (Ref. 106). Studies indicate that the CagA protein from *H. pylori*, which necessitates direct contact with host T cells through the T4SS, is associated with an increased risk of CRC and heightened inflammatory responses in individuals infected with CagA-positive strains (Refs 61, 107).

VacA: Unlike CagA, the virulence factor VacA appears to disseminate more easily beyond the stomach. Additionally, antigens such as (outer membrane protein) OMP (HP 1564), chaperonin GroEl and (*Helicobacter* cysteine-rich protein) HcpC have also been linked to an increased likelihood of CRC and are known to be released into the extracellular environment, much like VacA (Refs 51, 108, 109).

Lipopolysaccharides (LPS): A recent in vitro experimental study has revealed that the LPS of *H. pylori*, a principal component of its outer membrane, may disrupt the DNA repair mechanisms that are responsible for maintaining genomic stability in colonic cells. This disruption could lead to genotoxicity and subsequently influence the development of CRC (Ref. 110). Nitric oxide (NO) plays a crucial role in the stomach, acting as a messenger molecule that regulates blood flow, mucus production and gastric motility. It also contributes to protecting the gastric mucosa from ulcers and other damage. However, elevated levels of nitric oxide (NO) are produced by cells in response to *H. pylori* LPS, which is implicated in the genotoxicity associated with this bacterium. In colon cells, the carcinogenic potential of NO may be mediated by the inhibition of DNA repair enzymes and its interaction with pro-apoptotic proteins. These effects result the nitrosylation of specific cysteine and tyrosine residues, as demonstrated in other gastrointestinal cell types (Refs 111, 112). However, the specific biological roles of these antigens in the development of cancer, as well as the underlying mechanisms linking antibody responses to this group with an increased risk of CRC, have yet to be clarified.

#### Hypergastrinaemia

Hypergastrinaemia is characterized by elevated levels of the peptide hormone gastrin in the bloodstream, often resulting from various gastrointestinal conditions, including *H. pylori* infection (Ref. 113). The bacterium’s presence in the gastric epithelium can lead to chronic inflammation and damage to the gastric mucosa, which in turn affects gastric acid secretion (Ref. 104). The chronic infection of *H. pylori* can inhibit parietal cell function, leading to decreased gastric acid secretion and a compensatory increase in gastrin release (Ref. 114). The rise in gastrin levels has important consequences for gastrointestinal health. Elevated gastrin can enhance gastric acid secretion and promote the proliferation of gastric epithelial cells (Ref. 115). Studies have suggested that chronic hypergastrinaemia due to *H. pylori* infection may lead to changes in colonic mucosa and potentially increase susceptibility to colorectal neoplasia (Ref. 116). Recent research has established a significant association between gastrin levels and the up-regulation of inflammatory mediators, particularly cyclooxygenase 2 (COX-2) and IL-8 (Refs 117, 118). Moreover, the up-regulation of the inducible enzyme COX-2 following *H. pylori* infection has been implicated in the increased risk of CRC (Ref. 119). This association is supported by evidence linking COX-2 expression and tissue activity to the initiation and progression of CRC and various other neoplasms (Refs 120, 121). These elevations ultimately increase angiogenesis within the proliferating and mutagenic mucosal cells of the colon. This process is accompanied by reduced apoptosis, both of which are closely linked to carcinogenesis (Refs 121, 122).

### Indirect mechanisms

*Helicobacter pylori* may also influence host physiology through indirect mechanisms. These mechanisms do not directly engage with the host T cell but instead initiate cascades of physiological or immune responses that facilitate bacterial survival and pathogenesis. Indirect interactions often involve complex host–pathogen dynamics, including alterations in the host T-cell morphology, immune system modulation and changes to the gut microbiota. Each of these processes plays a critical role in shaping the infection outcome, and understanding them is crucial for developing therapeutic strategies.

#### Morphological shift in *H. pylori*: spiral to coccoid

The transformation of *H. pylori* from its spiral to coccoid form plays a significant role in its ability to evade the host’s immune system and resist antibiotic treatment, both of which may contribute to the development of CRC. One of the primary mechanisms by which *H. pylori* escape immune detection is through its coccoid form, known as the VBNC (viable but non-cultivable) state. This form can persist in adverse conditions, allowing the bacteria to survive despite the host’s immune response (Refs 123, 124). Studies indicate that coccoid forms can induce a humoral immune response similar to that of bacillary forms; however, they are less detectable by standard serological methods, which may complicate the identification of ongoing infections (Refs 125, 126).

A study by Jones et al. examined 176 colorectal specimens and found positive staining for *H. pylori* exclusively in the coccoid form, with no evidence of the typical spiral shape normally found in the stomach. Specifically, positive staining was observed in 1.7% of normal samples, 15.3% of adenomas and 16.9% of adenocarcinomas (Ref. 63).

The potential ability of *H. pylori* to evade the immune response might allow it to maintain chronic infections. These infections could lead to long-term mucosal inflammation, fostering a microenvironment that may be conducive to adenomas and malignant tumours in the colorectal region (Refs 104, 125, 127). Moreover, the coccoid form’s reduced ability to adhere to gastric epithelium and the lower antigenicity of these small particles may facilitate its survival. When antibiotics are administered, the bacillary forms are often targeted and eliminated; however, the coccoid forms may endure, potentially leading to the recurrence of infection. This persistence has been suggested to create a cycle of chronic inflammation and immune evasion that heightens the risk for CRC development, though more longitudinal clinical data are needed to confirm this mechanistic link (Refs 125, 128, 129).

#### Intracellular life of *H. pylori*

Although *H. pylori* is predominantly regarded as an extracellular pathogen, experimental evidence suggests it may possess the ability to penetrate epithelial cells (Ref. 130), macrophages (Ref. 131) and dendritic cells (Ref. 132), allowing it to survive and multiply within these cells. In adverse environmental conditions, it has been proposed that *H. pylori* may enter host T cells and remain dormant, possibly re-emerging when the environment becomes favourable again. This proposed intracellular life cycle may help explain how *H. pylori* could contribute to CRC. In vitro studies have shown that *H. pylori* can enter AGS cell lines (Refs 133–135) and persist in either spiral or coccoid forms within specialized membrane-bound vacuoles in the cytoplasm (Ref. 133). Numerous pathogenic bacteria such as *Legionella* (Ref. 136), *Mycobacterium* (Ref. 137) and *Pseudomonas* (Ref. 138) can endure adverse conditions by inhabiting eukaryotic cells. The ability of prokaryotes to live intracellularly is regarded as a crucial evolutionary development, facilitating their adaptation to diverse environmental habitats (Ref. 139).

An intriguing yet underexplored interaction between *H. pylori* and the yeast *Candida* has been identified, where the bacterium resides within the vacuoles of the yeast. Siavoshi et al. (Ref. 140) observed the rapid movement of bacterial-like bodies (BLBs) within the vacuoles of oral, gastric and vaginal yeasts (Ref. 141), suggesting that these yeasts may act as carriers for this pathogen. Given the ubiquitous presence of yeast throughout the digestive system, it is suggested that they might influence gut health (Refs 142, 143). Although studies have linked *Candida albicans* overabundance to IBD and CRC risk (Refs 144, 145), the direct role of yeast as a ‘Trojan Horse’ for *H. pylori* transmission to the colon is an emerging concept that requires more robust in vivo validation. Because yeasts are more resilient to environmental stresses than bacteria, they may help protect *H. pylori* in extra-gastric environments and facilitate its transfer to the human colon (Refs 140, 146). The intracellular life of *H. pylori* within yeast vacuoles may not only allow for nutrient acquisition but also promote its transmission within human populations. Similar to how amoebae have been proposed as training grounds for intracellular pathogens such as *Legionella* and *Mycobacterium* spp., yeast vacuoles might play a role in selecting important pathogenic features of *H. pylori* and enhancing its invasion capabilities. This interaction could contribute to the bacterium’s pathogenicity and its potential involvement in CRC, highlighting the need for further research into these complex relationships (Refs 147–149).

##### *Helicobacter pylori*-derived outer membrane vesicles

*Helicobacter pylori*-derived outer membrane vesicles (OMVs) represent a potential mechanism linking gastric infection to distal inflammatory and tumour-promoting effects in the colorectal microenvironment. The OMVs of *H. pylori*, characterized by their bilayer lipid membrane nanostructures, encapsulate a diverse array of bacterial components, including peptidoglycan (PG), LPS, outer membrane proteins, periplasmic proteins, various enzymes, DNA and toxins (Ref. 150). Recent studies suggest that OMVs from *H. pylori* may serve as an important aspect of virulence linked to the bacterium’s pathogenic mechanisms (Refs 151–153).

Evidence has shown that OMVs produced by *H. pylori* are implicated in disease progression by triggering inflammatory responses at both local and systemic levels, such as the modulation of neutrophil migration in areas beyond the stomach, thereby fostering a pro-inflammatory environment (Refs 154–156). They disrupt the regulation of immune responses by delivering biologically active substances into host T cells (Refs 154, 157). *Helicobacter pylori*-derived OMVs are detected by PRRs, such as (nucleotide-binding oligomerization domain-containing protein) NOD1, which may be involved in various host T-cellular processes, including invasion, autophagy, apoptosis and tumorigenesis (Refs 152, 158, 159). The absorption of *H. pylori* OMVs by epithelial cells can occur through either clathrin-dependent endocytosis or a clathrin-independent mechanism involving lipid rafts, and it significantly relies on the adhesins present in the OMVs.

Research has demonstrated that the presence of the VacA toxin may enhance the uptake of OMVs by host T cells (Refs 160, 161). The elements found in *H. pylori* OMVs can stimulate the release of both pro-inflammatory and anti-inflammatory cytokines, such as IL-6 and IL-10, and trigger an oxidative burst (Refs 162, 163). This process is facilitated by nicotinamide adenine dinucleotide phosphate (NADPH) oxidase, which becomes activated by various components of *H. pylori* OMVs, particularly HP-NAP (neutrophil-activating protein). This protein plays a crucial role in increasing β2 integrin expression and drawing leukocytes towards epithelial cells (Refs 164, 165). Additionally, these OMVs have the potential to trigger the degranulation of human eosinophils, resulting in the release of eosinophil cationic protein (ECP). This process raises the levels of intracellular adhesion molecule (ICAM)-1 on epithelial cells and enhances the expression of CD11b integrin on eosinophils (Refs 162, 166). Secreted OMVs from *H. pylori* can influence host transcription regulators, including NF-κB, MAPK and extracellular signal-regulated kinase (ERK), resulting in changes in gene expression that contribute to the secretion of cytokines such as IL-8, TNF-α and IL-1β, along with promoting cell growth and cancer progression (Refs 167, 168).

Several studies have examined the role of *H. pylori*-derived OMVs in extra-gastric diseases. For instance, previous research has suggested that *H. pylori*-derived OMVs can traverse biological barriers, reach brain tissue and contribute to the development of cognitive disorders. Astrocytes treated with OMVs secreted interferon-γ, which was dependent on NF-κB activation, leading to neurotoxicity (Refs 169, 170). Another investigation focused on the effects of *H. pylori* OMVs on liver cells, revealing that exosomes derived from OMV-contaminated hepatocytes showed increased expression of markers associated with fibrosis and stellate cell activation, including β-catenin, vimentin, α-SMA (smooth muscle actin) and (tissue inhibitor of metalloprotease) TIMP-1 (Ref. 171). Findings from an in vitro study by Hock et al. (Ref. 172) demonstrated that OMVs from *H. pylori* markedly stimulate COX-2 expression in peripheral blood mononuclear cells (PBMCs). This stimulation results in significantly increased levels of prostaglandin E2 and IL-10, which contribute to the inhibition of human T-cell responses. Emerging research suggests that CagA-positive *H. pylori*, unlike CagA-negative strains, contributes to the development of atherosclerosis by generating ROS through CagA-containing OMVs via the (Janus kinase signal transducer and activator of transcription) JAK-STAT3 signalling pathway (Refs 173, 174). The transmission of CagA proteins by OMVs to distant sites from the initial infection is associated with the promotion of Claudin-2 expression, alterations in cell structure and intestinal epithelial barrier dysfunction in chronic colitis and CRC (Refs 175, 176).

These findings suggest that OMVs may also contribute to CRC pathogenesis. Further investigation is needed to clarify the role of *H. pylori*-derived OMVs in colorectal tumorigenesis.

#### Autophagy process

The role of the autophagy pathway in CRC is complex, exhibiting both oncogenic and tumour-suppressive properties. Its effects are modulated by a variety of biological factors, such as genetic alterations, the specific type of tumour and its stage of development (Refs 75, 177, 178). Autophagy is a vital biological process characterized by the degradation and recycling of cellular components, enabling cells to maintain homeostasis and respond effectively to stressors. The formation of an autophagosome, characterized by its double-membrane structure, represents an essential step in the autophagy process. This structure arises from various morphological changes to the phagophore and associated membrane sources. When the autophagosome fuses with a lysosome, it leads to the degradation of targeted macromolecules and organelles (Refs 179, 180).

Autophagy regulation involves several signalling pathways, primarily 5′AMP-activated protein kinase (AMPK) and the mechanistic target of rapamycin (MTOR). Together, they inhibit ‘self-eating’ under favourable conditions and activate it during stressors like low energy, nutrient scarcity, hypoxia and the presence of pathogens (Ref. 181). AMPK facilitates the restoration of low energy levels within cells by suppressing the MTOR complex 1 (MTORC1) and activating UNC-like autophagy-activating kinase 1 (ULK1) through phosphorylation (Ref. 182). In stressful conditions, the inactivation of MTORC1 leads to the phosphorylation of ULK1 complex proteins, which collaborate with the class III phosphatidylinositol 3-kinase complex to initiate phagophore formation (Refs 183–185). The (antithymoglobulin) ATG5-ATG12-ATG16L1 complex facilitates the conversion of (microtubule-associated protein light chain) LC3-I to LC3-II, a critical step in selective autophagy that recruits damaged cargo via the adaptor sequestosome protein SQSTM1 (Refs 186, 187). Ultimately, autophagosomes mature through fusion with lysosomes, a process supported by proteins such as (lysosome-associated membrane protein) LAMP2 and RAB7. This fusion allows lysosomal enzymes to degrade the contents, recycling essential substrates back into the cytoplasm for cellular use (Refs 185, 188).

Different intracellular pathogens, such as *Yersinia pseudotuberculosis*, *Legionella pneumophila* and *H. pylori*, have developed various strategies to manipulate autophagy to their advantage. They do this by co-opting the membranes of autophagosomes to further their survival and proliferation (Ref. 189). Research has shown that chronic infection with *H. pylori* can lead to autophagy modulation that contributes to gastric cancer progression; this may also provide insights relevant to CRC (Refs 190, 191). Specifically, *H. pylori* can induce defective autophagy or inhibit it altogether, allowing the bacterium to thrive within epithelial cells and facilitate carcinogenesis (Refs 191, 192).

Autophagy impairment has been linked to aging (Ref. 193), neurodegenerative diseases (Ref. 194), cardiovascular disorders (Ref. 195), infections (Ref. 196) and cancers (Ref. 197). Numerous studies have indicated that the dysregulated expression of autophagy-related markers is linked to the advancement in CRC (Refs 198, 199). A study involving CRC patients revealed a significant correlation between LC3B expression and aggressive tumour phenotypes, indicating that autophagy may play a role in tumour promotion (Ref 70). In a separate study involving 155 CRC patients, the overexpression of Beclin1 – a key scaffold in autophagosome formation – was associated with increased tumour aggressiveness (Ref. 200). It is also revealed that ATG7 is essential for the survival of colon cancer cells, and its inhibition triggers cell death by activating apoptotic pathways (Ref. 201).

Research has demonstrated that *H. pylori*, along with the OMVs it produces (Refs 151, 152), which are known to carry virulence factors, can disrupt T-cellular mechanisms (Refs 202, 203). In the investigation by Yang et al. (Ref. 199), it was demonstrated that *H. pylori* significantly up-regulates Beclin1 expression and increases the LC3-II/I ratio while concurrently reducing p62 protein levels in human colon carcinoma cells. Furthermore, the activation of autophagy in colon cells leads to elevated levels of the cell proliferation marker Ki67 (discovered in Kiel, Germany) and the apoptosis inhibitor (B-cell lymphoma-2) Bcl-2, indicating that *H. pylori* infection is associated with enhanced cellular proliferation and suppressed apoptosis in CRC (Ref. 199). Recent studies have highlighted the potential interaction between *H. pylori* infection, autophagy and apoptosis, suggesting that this connection occurs through the binding of Bcl-2 and Bcl-xL to Beclin1, thereby influencing autophagic processes (Ref. 204). During *H. pylori* infection, VacA leads to an elevated autophagic flux index, particularly reflected in increased levels of LC3-II (Refs 184, 192, 205). Findings from Lai et al. (Ref. 206) suggest that cholesterol glucosyltransferase (CGT) from *H. pylori*, which converts host cholesterol to cholesterol-α-glucosides, enhances the survival of this bacterium in J774A.1 macrophage cells promote autophagy by increasing the levels of LC3-II, ATG12 and Beclin1. Additionally, CagA-positive strains can alter autophagy through the c-Met-PI3K/Akt–mTOR signalling pathway, resulting in the release of pro-inflammatory cytokines such as IL-1β, TNF-α and IL-8 (Ref. 202).

Further research is needed to clarify how *H. pylori*-induced autophagy dysregulation contributes to colorectal tumour biology. Understanding these mechanisms may support the development of therapeutic strategies aimed at restoring autophagic balance and potentially reducing CRC-related pathogenic effects.

#### Intestinal immune responses induced by *H. pylori*

*Helicobacter pylori* modulates both innate and adaptive immune responses, contributing to the progression of infection-associated disease (Ref. 207). Through its array of virulence factors, *H. pylori* not only evade initial immune responses but also subtly modulates T-cell activity and antigen presentation (Ref. 208). This allows it to persist within the host and establish chronic infection, all while circumventing the immune defences. This evasion strategy is a hallmark of *H. pylori*’s ability to manipulate host immune processes, leading to chronic infection and the promotion of a pro-inflammatory environment, which may increase the risk of CRC (Refs 29, 209).

One key aspect of *H. pylori* infection is its influence on T-cell populations, particularly CD4^+^ and CD8^+^ T cells, which contribute to inflammation. The bacterium induces a mixed Th1 and Th17 response in the gastric mucosa, with the release of pro-inflammatory cytokines like interferon gamma (IFN-γ) and IL-17A, which are critical to the pathology of gastritis (Ref. 210). Dendritic cells (DCs) that encounter *H. pylori* up-regulate markers such as CD83 and CD86, which in turn drive a strong Th1 response. IL-23-producing DCs also stimulate Th17 responses, thereby intensifying inflammation (Refs 211, 212). Experimental studies have shown that both *H. pylori*-specific CD4^+^ T cells and the sustained secretion of IFN-γ are essential for the development of gastritis, as mice lacking these components fail to exhibit significant gastric inflammation. Furthermore, regulatory T cells (Tregs) are crucial in maintaining immune homeostasis, counter-acting the inflammatory Th1/Th17 response and enabling *H. pylori*’s persistence in the host (Refs 213, 214).

Research has also indicated that Peyer’s patches (PPs) in the small intestine are vital for priming naive CD4^+^ T cells against *H. pylori* antigens. PPs can engulf the coccoid form of *H. pylori*, allowing DCs to capture its antigens and subsequently activate CD4^+^ T cells (Ref. 215). Studies have shown that mice with mutations in the adenomatous polyposis coli (APC) gene develop a higher tumour burden when infected with *H. pylori* compared to non-infected controls. This increased tumorigenesis is associated with an imbalance between pro-inflammatory and regulatory T cells within the intestinal epithelium (Ref. 216). Furthermore, *H. pylori* infection has been linked to the activation of pro-carcinogenic signalling pathways such as STAT3 in both intestinal and colonic epithelium. The persistent pro-inflammatory immune response driven by *H. pylori* infection contributes to the development of CRC through mechanisms that include increased gut barrier permeability and subsequent antigen influx (Refs 217, 218).

**Figure 3.**
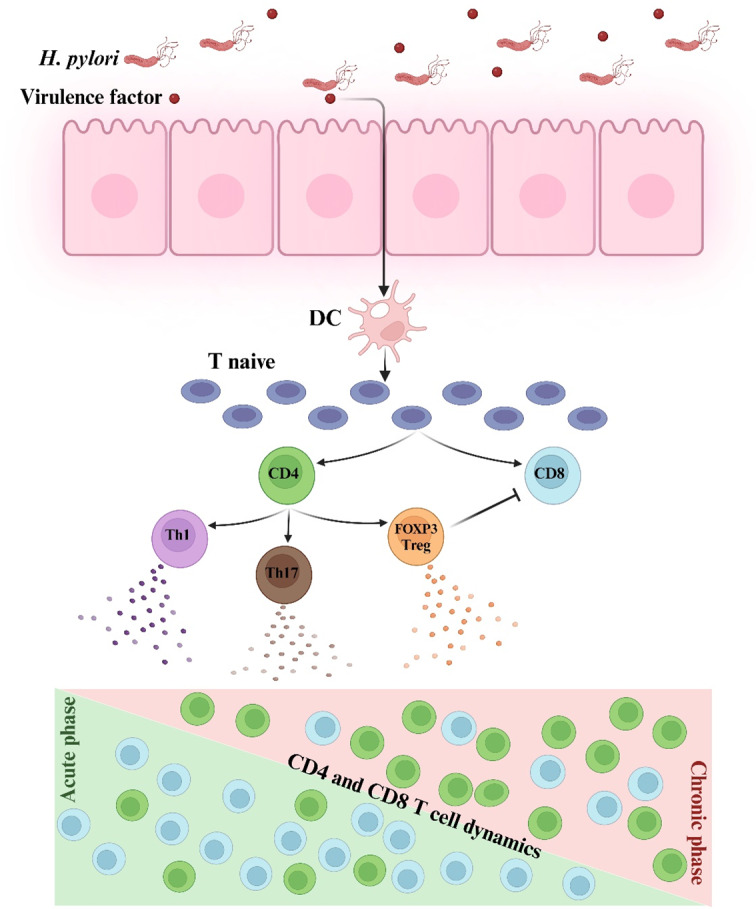
Dynamics of CD4^+^ and CD8^+^ T-cell responses during *Helicobacter pylori* infection. *Helicobacter pylori* trigger immune responses by activating dendritic cells (DCs), leading to CD4^+^ T-cell differentiation and CD8^+^ T-cell activation. The acute phase features a robust CD8^+^ T-cell response, while the chronic phase is marked by CD4^+^ T-cell dominance, facilitating immune evasion and persistent inflammation, which drives disease progression and may contribute to the development of CRC. Created by biorender.com. Figure 3. long description.

Recent findings have highlighted a dynamic role for CD8^+^ T cells in *H. pylori* infection. At the early stages of infection, tissue-resident CD8^+^ T cells respond to the bacterial CagA antigen by producing IFN-γ, contributing to the initial immune response. As illustrated in Figure 3, the transition from early to chronic *H. pylori* infection is accompanied by a decline in CD8+ T-cell populations and a progressive predominance of CD4+ T cells, indicating a shift in the immune landscape during persistent infection (Refs 219, 220). Interestingly, studies have shown that CD8^+^ T-cell infiltration increases in the gastric mucosa when CD4^+^ T cells are absent. Such changes correlate with more severe disease manifestations. This suggests that CD4^+^ T cells may play a regulatory role in controlling CD8^+^ T-cell activity during *H. pylori* infection (Refs 221, 222). In terms of cancer prognosis, tumours with higher levels of CD8^+^ T-cell infiltration are associated with better outcomes due to the role of these cells in controlling tumour growth and their response to immunotherapies (Refs 223–225). However, the specific impact of *H. pylori* on CD8^+^ T-cell function during carcinogenesis remains under investigation. There is evidence suggesting that the bacterium’s presence might suppress tumour-specific CD8^+^ T-cell responses through mechanisms involving increased numbers of (forkhead box protein) FoxP3^+^/IL-17^+^ T cells in the intestines, which could impair effective anti-tumour immunity (Ref. 226).

Recent studies have also demonstrated that *H. pylori* can dampen systemic CD8^+^ T-cell responses, potentially reducing the effectiveness of cancer immunotherapies. In models such as MC38 colon adenocarcinoma and azoxymethane/DSS-induced tumours, *H. pylori* infection was found to increase tumour size and reduce the effectiveness of treatments targeting (cytotoxic T-lymphocyte-associated protein) CTLA-4 and (programmed cell death ligand) PD-L1 pathways. This is attributed to a reduction in the activation and proliferation of tumour-specific CD8^+^ T cells, driven by altered dendritic cell function in infected mice (Refs 220, 227).

Overall, *H. pylori* have a profound influence on the immune system, particularly through its modulation of CD4^+^ and CD8^+^ T cells. By affecting CD4^+^ T-cell differentiation, the bacterium plays a critical role in disease pathogenesis, including the heightened risk of CRC. Additionally, *H. pylori* may dampen tumour-specific CD8*+* T-cell responses, thus hindering the effectiveness of cancer immunotherapy and potentially influencing colon carcinogenesis through immune cell dynamics.

#### *Helicobacter pylori* and non-coding RNAs interactions

For decades, scientific inquiry has predominantly centred on the mere 2% of the human genome responsible for encoding proteins (Refs 228, 229). However, recent advancements have illuminated the significance of the remaining 98%, previously dismissed as non-functional ‘junk’ DNA. This vast segment of our genetic material encompasses non-coding RNAs (ncRNAs), which are now recognized as pivotal players in a multitude of biological processes, including growth, development and organ function. Non-coding RNAs are categorized into two main types: small non-coding RNAs, which include microRNAs (miRNA), and long non-coding RNAs (lncRNAs) (Refs 230). The miRNA are small, single-stranded non-coding RNAs, approximately 22 nucleotides long, encoded by genes within the genome. They play a crucial role in regulating gene expression by binding to target messenger RNAs (mRNAs) and specifically interacting with the 3′-untranslated region (UTR). This interaction can lead to the degradation of the mRNA or inhibition. Consequently, it reduces the expression of specific target genes (Refs 231, 232). The regulation of gene expression by miRNAs is essential for various biological processes, including tumour development, metastasis and drug resistance (Refs 233–235). On the other hand, lncRNAs are defined as RNA molecules longer than 200 nucleotides (Ref. 236). While they do not have the capacity to encode proteins, lncRNAs fulfil a variety of complex regulatory roles (Ref. 237). They can impact a wide array of biological processes, such as transcription, translation, cellular architecture, cell cycle progression, apoptosis and the maintenance of stem cell pluripotency (Refs 236, 238–242).

*Helicobacter pylori* influence on miRNAs is well-established in gastric oncogenesis (Ref. 243); however, its impact on the colonic ncRNA profile is an emerging area of research. The study by Zhong et al. (Ref. 82) demonstrated that CagA-positive *H. pylori* promotes autophagy in colon cancer cells. This occurs through the down-regulation of miR-125b-5p, which in turn affects LC3B-II/LC3B-I and Beclin-1 levels. The suppression of miR-125b-5p, a recognized tumour suppressor, contributes to increased cell proliferation, invasion and enhanced autophagic processes – key mechanisms in cancer development. The findings reveal that overexpression of the CagA protein from *H. pylori* leads to decreased levels of miR-125b-5p, while restoration of miR-125b-5p expression effectively reverses these effects, inhibiting both autophagy and cell invasion. These results suggest a potential link where *H. pylori* infection might not only play a direct role in the initiation of CRC but also modulate critical non-coding RNAs like miR-125b-5p, thereby linking microbial infection to tumorigenesis (Ref. 82). Several other miRNAs have also been implicated in *H. pylori*-associated cancer progression, supporting a role for this bacterium in gene regulation. For instance, miR-21 is frequently up-regulated during *H. pylori* infection and has been proposed to promote cancer progression by targeting tumour suppressor genes such as RECK (reversion-inducing-cysteine-rich protein with kazal motifs), TGF-β1 and TGF-βR2 (transforming growth factor), thereby facilitating cell proliferation and invasion (Refs 244, 245). Similarly, miR-155 is up-regulated in response to the infection, potentially contributing to inflammation and tumorigenesis by regulating cytokine production and targeting the (Sma- and Mad- related protein) SMAD2 gene, which is involved in tumour growth and immune response modulation (Ref. 246). In contrast, *H. pylori* down-regulate specific tumour-suppressive miRNAs, such as let-7b, which normally inhibits pathways critical for inflammation and cancer development. The reduction of let-7b expression is hypothesized to lead to enhanced activity of pro-inflammatory genes like TLR4, NF-κB, COX-2 and IL1B, promoting a chronic inflammatory environment that facilitates tumorigenesis (Refs 247, 248). Another important miRNA related to *H. pylori* infection, miR-146a, inhibits components of the immune response, such as (interleukin 1 receptor associated kinase) IRAK1 and (caspase recruitment domain) CARD10, while promoting tumour-supportive pathways, including COX2 and PTGS2, further linking chronic inflammation with cancer progression (Refs 249, 250). Moreover, miR-200b and miR-200c, which are also up-regulated, play crucial roles in promoting epithelial–mesenchymal transition (EMT), a process essential for cancer metastasis. By down-regulating targets like (zinc-finger E-box-binding homeobox) ZEB1 and IL6, these miRNAs enable cancer cells to gain migratory and invasive properties (Refs 247, 251). ZEB1 is closely associated with cagPAI^+^ *H. pylori* strains (Ref. 251) and has been linked to poor prognostic outcomes in CRC, highlighting its role in promoting EMT. By facilitating the invasive properties of cancer cells through pathways influenced by *H. pylori*, ZEB1 may serve as a pivotal player in the progression of CRC (Refs 252, 253). Xie et al. (Ref. 254), revealed a significant association between lncRNA ZFAS1 and *H. pylori* infection. This link extends to lymph node metastasis, advanced TNM stage and poor overall survival in CRC patients. Additionally, functional assays demonstrated that inhibiting (zinc finger antisense) ZFAS1 markedly diminished both the proliferation and invasion capabilities of CRC cells in vitro and in vivo, ultimately leading to a reduction in tumour growth. Bioinformatics assessments and luciferase reporter assays confirmed a direct interaction between ZFAS1 and miR-484. Furthermore, the study indicated that employing a miR-484 inhibitor could reverse the tumour-suppressing effects observed with ZFAS1 knockdown. Consequently, ZFAS1 is characterized as an oncogene associated with *H. pylori*, is regulated by the miR-484 pathway, which plays a pivotal role in CRC tumorigenesis (Ref. 254).

Collectively, the alterations in miRNAs, their target genes and lncRNA profiles induced by *H. pylori* infection are increasingly recognized as a possible molecular bridge between initiation of tumorigenic pathways and CRC, highlighting their significance in the pathogen’s oncogenic mechanisms. However, further clinical studies are necessary to confirm whether these non-coding RNA dysregulations observed in experimental models translate directly to the human colonic landscape during chronic *H. pylori* infection.

#### Changes in gut microbiota communities induced by *H. pylori* infection

Early research has established a significant connection between microorganisms and the development of CRC, with studies dating back to the 1960s exploring how gut microbiota influences CRC susceptibility. Notably, a pivotal study from 1967 demonstrated that intestinal microorganisms were essential in mediating the carcinogenic effects of cycasin in conventional rats, while germ-free rats exhibited no such cancer development (Ref. 255). Further investigations revealed that when both germ-free and conventional rats were exposed to the carcinogen 1,2-dimethylhydrazine, a staggering 93% of conventional rats developed colonic tumours compared to only 21% of their germ-free counterparts (Refs 256, 257). The role of gut microbiota in colorectal carcinogenesis has gained renewed attention, particularly in relation to *H. pylori* infection. Table 2 summarizes the reported effects of *H. pylori* infection on gut microbiota composition. Recent findings indicate that *H. pylori* infection not only disrupts the balance of commensal bacterial species within the gastric mucosa but also induces substantial changes in intestinal microbial communities. This shift towards dysbiosis is associated with various gastrointestinal disorders and may play a role in carcinogenesis (Refs 259, 262, 267–272).

**Table 2.** The effects of *Helicobacter pylori* infection on gut microbiota compositions Table 2. long description.

Study design	Study model	Study details		Statistical methods used	Microbiota changes	Potential functional implications	References
		Sample size	Methodology				
Experimental design	Animal model	29 Mongolian gerbils: –12 uninfected controls –11 infected with wild-type *H. pylori* –6 infected with mutant *H. pylori* (lacking cagY)	PCR-DGGE and sequence analysis	Mann–Whitney U test	↑ *Escherichia coli*, *Enterococcus* spp., *Bacteroides/Prevotella* spp., *Akkermansia*	Promotion of intestinal inflammation and potential mucosal barrier impairment	(Ref. 258)
Experimental design	Animal model	28 Apc-mutant mice: –10 Uninfected controls –18 *H. pylori*-infected 8 WT mice 8 Germ-free mice	–Flow cytometry –Immunohistochemistry –RNA sequencing	–Mann–Whitney *U* test –Student’s *t* test –Kruskal-Wallis test –One-way ANOVA –Tukey’s multiple comparisons	↑ *Akkermansia*, *Ruminococcus* spp.	Induction of pro-carcinogenic immune responses and acceleration of tumour progression	(Ref. 216)
Experimental design	Animal model	≈12–20 Apc+/1638 N mice ≈12–20 C57BL/6 mice	–Meta-genomic sequencing –Deephage	–Shannon and Chao1 index –PERMANOVA –ANCOM-BC	↑ Caudoviricetes, Malgrandaviricetes and Faserviricetes	Viral-mediated gut homeostasis disruption and transfer of virulence genes	(Ref. 69)
Cross-sectional study	Human model	56 school-aged children (6–12 years): –28 with *H. pylori* *–*28 without *H. pylori*	PCR amplification	–T-test –Kruskal-Wallis – χ ^2^ test – Fisher’s exact test	↑ Proteobacteria, *Clostridium*, Firmicutes and *Prevotella*	Weakening of the gastric acid barrier and promotion of intestinal dysbiosis	(Ref. 259)
Cross-sectional study	Human model	159 students: –80 with *H. pylori* –79 without *H. pylori*	16S rRNA sequencing	–Welch’s *t* test –PERMDISP	↑ *Prevotella, Collinsella* ↓*Subdoligranulum*	Reduction in butyrate-producing bacteria and SCFA metabolism; alteration of the gastric immune microenvironment and promotion of local inflammatory responses.	(Ref. 260)
Observational study	Human model	17 adult participants: –10 with *H. pylori* –7 without *H. pylori*	16S rRNA sequencing	–Wilcoxon signed rank test –ANOVA –PCoA	↑ Proteobacteria, Actinobacteria, Acidobacteria and *Alistipes* ↓ *Subdoligranulum*, *Lachnoclostridium*	Gut microbiota homeostasis disruption and reduction of beneficial commensal-mediated anti-inflammatory signalling.	(Ref. 261)
Population-based cohort study	Human model	424 adult participants: –212 with *H. pylori* –212 without *H. pylori*	16S rRNA sequencing	–Shannon index –PERMANOVA –Mann–Whitney tests –‘R’ function lm	↑ *Prevotella*, *Haemophilus*, *Holdemanella*, Betaproteobacteria, *Alisonella* and *Howardella* ↓Bacteroidetes, *Pseudoflavonifracto, Fusicatenibacter*, *Alistipes*, *Parasutterella* and *Barnesiella*	Promotion of systemic low-grade inflammation and potential contribution to extra-gastroduodenal metabolic disorders	(Ref. 262)
Randomised, double-blind, placebo-controlled trial	Human model	66 adult participants: –19 without *H. pylori* –23 in probiotic group –24 in placebo group	–Fluorescence in situ hybridization (FISH)	–Wilcoxon signed-rank test –Two-way Student’s t-test	↑ Total number of anaerobes and *Clostridia*	Disruption of intestinal homeostasis and increased susceptibility to antibiotic-associated gastrointestinal side effects during eradication therapy	(Ref. 263)
Open-label, randomized clinical trial	Human model	105 adult participants: –70 with *H. pylori* –35 without *H. pylori*	16S rRNA sequencing	–Wilcoxon rank sum test –ANOVA –Kruskal-Wallis test –LEfSe	↑ *Sphingomonas spp.*, *Turicibacter spp.* ↓ *Nitrospirae*, *Bacteroides plebeius*	Impaired metabolic function and increased gastrointestinal symptoms	(Ref. 264)
Observational study	Human model	313 adult participants: –128 with *H. pylori* –185 without *H. pylori*	Meta-genomic sequencing	–Wilcoxon test –Chi-square test –ROC analysis	↑ *Prevotella copri*, *Enterobacter cloacae* and *Klebsiella pneumoniae* ↓ *Sutterella wadsworthensis*, *Bacteroides vulgatus* and *E. coli*	Impaired metabolic function and systemic inflammation	(Ref. 265)
Observational cohort study	Human model	428 adult participants: –214 with *H. pylori* –214 without *H. pylori*	16S rDNA sequencing	–Chi-square test –Student’s t-test –Wilcoxon’s matched-paired signed-rank test –Shannon and Chao1 – PERMANOVA	↑ *Actinomyces*, *Gemella*, *Streptococcus*, *Haemophilus*	Oral-gut microbial translocation and mild disruption of gut microbial compartmentalization associated with low-grade inflammatory potential	(Ref. 266)
Observational study	Human model	60 adult participants: –12 with *H. pylori* –48 without *H. pylori*	16S rRNA and ITS2-based microbial profiling	–ANOVA –Shannon and Bray-Curtis indices –PERMANOVA	↑ *Succinivibrio*, *Coriobacteriaceae*, *Turicibacter*, Enterococcaceae, *Desulfovibrio*, Rikenellaceae, Enterococcaceae and *Candida glabrata*	Potential oral-gut microbial translocation with low-grade inflammatory potential	(Ref. 267)
Randomized clinical trial	Human model	60 Adult participants: –20 Triple therapy group –20 Triple therapy + probiotics group –20 Healthy control	16S rDNA sequencing	–Parametric paired-samples t-tests –Pearson’s correlation coefficient	↓ *Actinobacteria*, *Gemmatimonadetes*, *Nitrospirae*, *Chlorobi*, *Thermi*, WS3, *Caldithrix*	Impaired host immune regulation and reduced resistance to intestinal pathogens	(Ref. 268)
Cross-sectional study	Human model	60 Adult participants: –40 with *H. pylori* –20 without *H. pylori*	16S rRNA sequencing	–Wilcoxon signed-rank test –Mann–Whitney U test –Spearman’s correlation coefficient –Multivariate regression analysis	↑Bacteroidetes, ↑Bacteroidetes/Firmicutes ratio ↓ Firmicutes and Actinobacteria	Alteration of host energy metabolism, potentially contributing to a lower body mass index (BMI)	(Ref. 269)
Observational design	Human model	47 adult participants –8 past *H. pylori* Infection –24 current *H. pylori* Infection –15 without *H. pylori*	16S rRNA sequencing	–Shannon index –Mann–Whitney test –PCoA –ANOSIM –Welch’s t-test	↑Bacteroidetes, Firmicutes, Proteobacteria	Development of an intestinal pro-inflammatory environment	(Ref. 270)

A study by Heimsat et al. (Ref. 258) on Mongolian gerbils infected with *H. pylori* revealed significant changes in the large intestinal microbiota after 14 months of infection. Notably, there was an increase in *Escherichia coli*, *Enterococcus* spp. and *Bacteroides*/*Prevotella* spp., along with a rise in *Akkermansia*, a mucus-degrading species linked to compromised intestinal barrier function. Alongside these microbiota changes, a pro-inflammatory response was observed, characterized by an increase in CD3^+^ T-cell infiltration within the colonic tissue. These microbiota changes may be driven by hypochlorhydria and hypergastrinaemia induced by *H. pylori*, which facilitate the passage of acid-sensitive bacteria into the distal gastrointestinal tract. *H. pylori*’s virulence factor CagA also plays a role in promoting inflammation and disrupting the epithelial barrier, further contributing to microbiota imbalances (Ref. 258).

In wild-type mice, *H. pylori* infection induced microbial shifts in faecal, cecal and ileal samples, with significant changes in Turicibacteraceae, Erysipelotrichaceae and Desulfovibrionaceae families (Ref. 273). A study on *Apc*-mutant mice infected with *H. pylori* showed an overgrowth of mucosa-destroying species, particularly *Akkermansia* and *Ruminococcus* spp., leading to intestinal barrier disruption and potential tumour promotion. Interestingly, faecal transplantation from *H. pylori*-infected tumour-prone mice to germ-free recipients caused reduced Treg levels and enhanced STAT3 activation, speeding up tumour development compared to non-infected stool recipients (Refs 216, 274). These findings underscore the critical role of *H. pylori* in reshaping the gut microbial community and its implications for CRC development.

In children’s studies infected with *H. pylori*, there was an increase in various bacterial populations within their gut microbiota, including *Proteobacteria*, *Clostridium*, *Firmicutes*, *Prevotella* and *Proteus* (Refs 259, 260). He et al. reported that patients infected with *H. pylori* exhibited an increased frequency of *Alistipes*, along with a decrease in *Lachnoclostridium* (Ref. 261). Also, in a large cohort study, individuals infected with *H. pylori* showed an increase in *Prevotella*, *Parasutterella*, *Holdemanella*, *Betaproteobacteria*, *Alisonella* and *Howardella*. The level of *H. pylori* antigen had a stronger impact on changes in gut bacteria than factors like age or sex. It was also negatively correlated with beneficial bacteria such as *Bacteroides*, *Fusicatenibacter*, *Alistipes* and *Barnesiella* (Ref. 262). Using shotgun meta-genomic sequencing, Wang et al. (Ref. 265) found a correlation between *H. pylori* and the increased proliferation of pathogenic bacteria, such as *Prevotella copri*, *Enterobacter cloacae* and *Klebsiella pneumoniae*. In contrast, they observed a decrease in the levels of *Sutterella wadsworthensis*, *Bacteroides* (*Phocaeicola*) *vulgatus* and *E. coli* associated with *H. pylori* infection, suggesting that these bacteria may play a role in modulating immune responses.

Additionally, *H. pylori* infection significantly impacts the fungal and viral populations in the gut (Refs 69, 267). In the research conducted by Dash et al. (Ref. 267), a microbial profiling analysis utilizing 16S rRNA and ITS2 was performed on stool samples from adults infected with *H. pylori*. The analysis revealed not only changes in bacterial populations (increase of Succinivibrio, Coriobacteriaceae, Enterococcaceae, Rikenellaceae) but also a significant increase in *Candida glabrata* levels and various unclassified fungi. Luo et al. (Ref. 69) observed a striking transformation in the intestinal viral community of mice with *H. pylori*-induced CRC. Specifically, the infection led to an increased frequency of viral operational taxonomic units (vOTUs) from the Caudoviricetes, Malgrandaviricetes and Faserviricetes classes. These changes in viral populations suggest an intricate interplay between viral dynamics and cancer progression (Ref. 69). The connection between these microbial shifts and disease progression is further illuminated by studies linking temperate phages (which can replicate either lysogenically as prophages or lytically by producing new viral particles) to the worsening of dysbiosis-related diseases such as IBD and CRC. It is thought that the rise in these phages contributes to the disruption of the gut microbiome through immune responses triggered by bacterial lysis and the release of phage progeny (Refs 69, 275, 276). This process can lead to the release of PAMPs and damage-associated molecular patterns (DAMPs), which in turn trigger inflammatory cytokine production, including IL-12, IL-6, IL-10 and IFN-γ-induced protein 10. These inflammatory signals can exacerbate intestinal inflammation and dysbiosis, contributing to the progression of diseases like CRC and IBD (Refs 277–279).

Together, these findings indicate that *H. pylori* infection can alter gut microbial communities in ways that may influence intestinal inflammation and CRC risk. Effective management of *H. pylori* may therefore have implications beyond gastric health, though this possibility requires further clinical investigation.

## Translational implications: probiotics as a therapeutic and preventive strategy

While the protective effects of *H. pylori* eradication against gastric cancer are well documented, the impact of this intervention on the risk of subsequent CRC remains ambiguous (Ref. 280). Currently, there is a scarcity of studies investigating the relationship between *H. pylori* eradication and CRC development, highlighting a significant gap in our understanding of this potential association. In this regard, the study conducted by Guo et al. (Ref. 128), which involved a follow-up of about 10 years, found that treatment for *H. pylori* significantly reduces the rate of CRC among patients. This suggests that treating *H. pylori* may provide additional benefits in lowering the risk of CRC. A follow-up investigation of a randomized controlled trial focused on the treatment of *H. pylori* and its impact on gastric cancer revealed that eradicating *H. pylori* is linked to a decreased long-term risk of death specifically from CRC. This association remained significant even after adjusting for initial gastric tissue conditions, with a hazard ratio: 0.25, 95% CI: 0.07–0.89 (Ref. 281). Research has demonstrated that antibiotic treatments for *H. pylori* lead to a decrease in the alpha diversity of gut microbiota. Following the successful eradication of *H. pylori*, there is an increase in bacteria that produce short-chain fatty acids, which have been associated with anti-cancer effects against CRC (Refs 267, 282, 283).

The standard treatment for *H. pylori* infection conventionally consists of antibiotic-based eradication therapy combined with acid suppression, commonly using a proton pump inhibitor together with two antibiotics, such as clarithromycin plus amoxicillin or metronidazole (Refs 284, 285). Although eradication may provide important clinical benefits, it also has potential drawbacks. Antibiotic-based therapy can temporarily disturb gut microbiota composition, reduce beneficial commensal bacteria and cause gastrointestinal side effects such as diarrhoea, abdominal discomfort and antibiotic-associated dysbiosis (Refs 267, 282). In addition, widespread or repeated eradication therapy may contribute to antibiotic resistance, particularly resistance to clarithromycin and metronidazole, which can reduce treatment success and limit future therapeutic options (Refs 284, 285). Therefore, eradication should not be viewed as a universally risk-free intervention. Treatment decisions should consider the clinical indication, individual cancer risk, previous antibiotic exposure, local resistance patterns and possible microbiome-related consequences.

**Figure 4.**
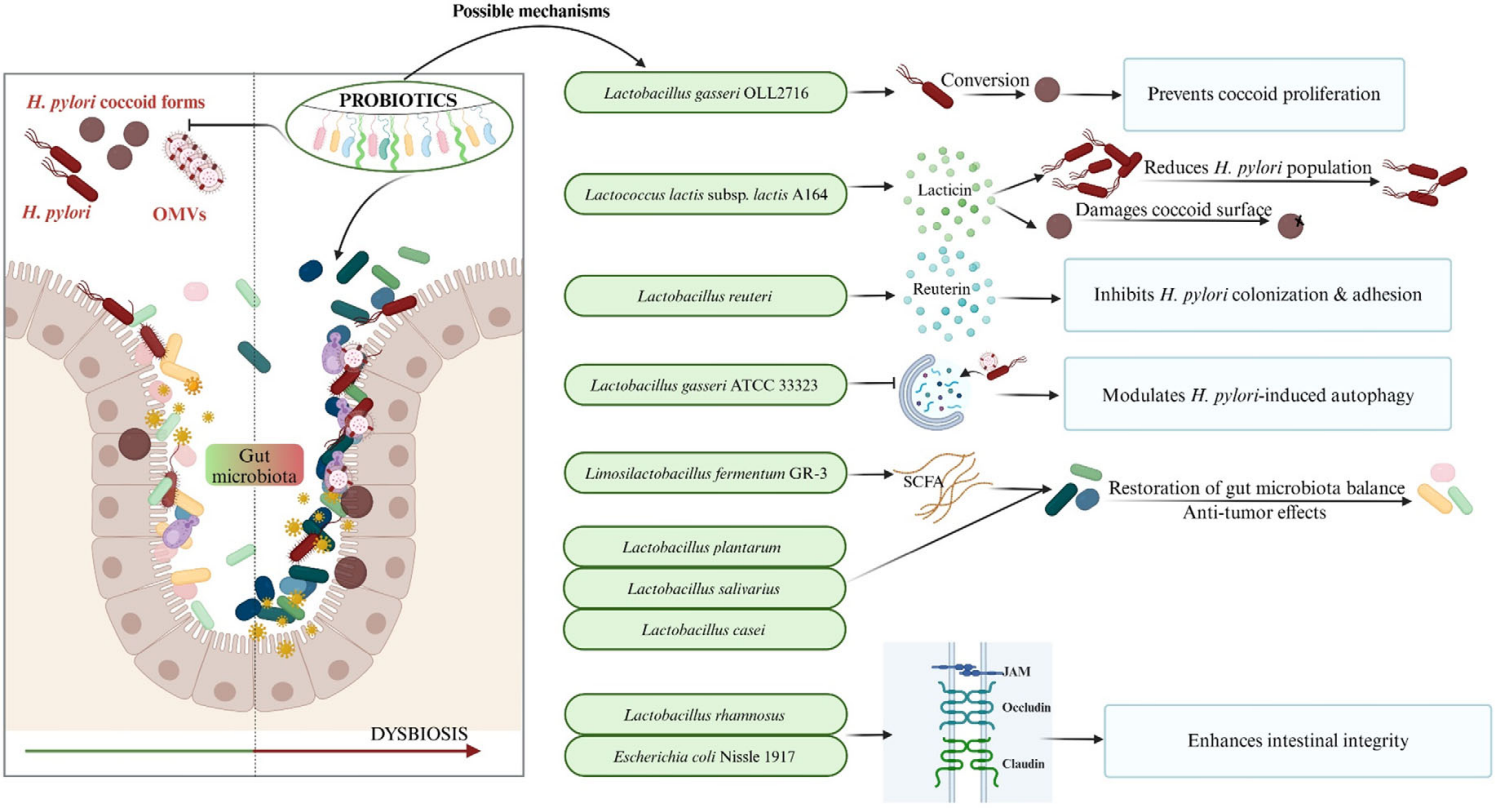
Schematic of probiotic mechanisms in modulating *Helicobacter pylori* infection and gut microbiota in CRC prevention. Certain probiotics inhibit *H. pylori* infection by directly targeting both the spiral and coccoid forms of the bacterium and its secreted components. Other probiotics mitigate the harmful effects of *H. pylori* by restoring dysregulated microbiota and enhancing the integrity of intestinal epithelial tight junctions. Created by biorender.com. Figure 4. long description.

The rise of antibiotic resistance in *H. pylori* and other pathogens has led to increased interest in alternative approaches. This has intensified interest in microbiome-based strategies for disease prevention and treatment. Studies have highlighted the potential of beneficial microorganisms to promote health and protect against various conditions (Refs 286, 287). Figure 4 summarizes the proposed probiotic-related mechanisms relevant to CRC prevention and management, including immunomodulation, anti-inflammatory effects and suppression of tumour-promoting processes. Importantly, much of this evidence is derived from preclinical models and extrapolation to human CRC prevention requires careful consideration.

Fujimura et al. (Ref. 288) showed that simultaneous cultivation of *H. pylori* and the probiotic *Lactobacillus gasseri* OLL2716 for 24 hours resulted in a noticeable transformation of the bacteria from their spiral form to a coccoid shape. Furthermore, attempts to re-cultivate the coccoid form on sheep blood agar plates were unsuccessful. In another study, researchers induced the coccoid form of *H. pylori* and examined the effects of *Lactococcus lactis* subsp. *lactis* A164, a probiotic isolated from kimchi. They discovered that this probiotic produces lacticin and not only reduces the microbial population of *H. pylori* but also damages the surface of its coccoid form (Ref. 289). The shift towards a non-cultivable state suggests functional attenuation of *H. pylori* and underscores the relevance of targeting this persistent morphology, given its proposed role in chronicity and microenvironmental alterations associated with colorectal carcinogenesis. Mukai et al. (Ref. 290) emphasize the potential of *Lactobacillus* (*Limosilactobacillus*) *reuteri* to inhibit *H. pylori* by preventing its adhesion to gastric glycolipid receptors. By interfering with this early step of bacterial attachment, *Lactobacillus reuteri* may also have the potential to influence infection-associated inflammatory pathways; however, whether such effects could extend beyond the stomach and contribute to colorectal carcinogenesis remains speculative and requires further investigation. In vivo investigations have demonstrated that the intake of yogurt enriched with diverse strains of *L. gasseri* can alleviate symptoms associated with dyspepsia, mitigate inflammation of the gastric mucosa and enhance the efficacy of conventional triple therapy in individuals infected with *H. pylori* (Refs 291–293). *Lactobacillus gasseri* ATCC 33323 strains affect the expression of several factors, including IL-8, β-catenin and BCL-2. They also modulate the integrin subunit α5β1 in epithelial cells exposed to clinical strains of *H. pylori*. These strains also inhibit autophagy through the ATG16L1-NOD1 pathway, which is triggered by *H. pylori*’s OMVs, thereby helping to protect host T-cells from the activity of *H. pylori* (Refs 152, 294). Although these findings are primarily derived from gastric models of *H. pylori* infection, the observed modulation of inflammatory signalling, apoptosis-related pathways and β-catenin may suggest a potential, albeit indirect, relevance to CRC.

Probiotics, in addition to their advantageous effects on *H. pylori* – an organism linked to CRC development – demonstrate significant potential in restoring gut dysbiosis associated with CRC through a variety of mechanisms. For instance, *Limosilactobacillus fermentum* GR-3, sourced from traditional fermented foods, has been shown to effectively reduce colitis and lower tumour occurrence in a mouse model of CRC induced by azoxymethane and dextran sulphate sodium (AOM/DSS). This strain enhances the modulation of the gut microbiome by promoting the growth of beneficial short-chain fatty acid (SCFA) producers, such as the Lachnospiraceae NK4A136 group, while simultaneously inhibiting pro-inflammatory bacteria like Bacteroides (Ref. 295). Probiotics such as *Lactobacillus* (*Lacticaseibacillus*) *rhamnosus* and *E. coli* Nissle 1917 play a vital role in microbiota composition. They strengthen intestinal epithelial integrity by promoting the expression of tight junction proteins, such as claudin-1 and occludin, to prevent CRC. These beneficial microorganisms contribute to maintaining gut barrier function, effectively reducing inflammation and protecting against oncogenic pathogens such as *H. pylori*, whose translocation can lead to cancer development (Refs 296, 297). In the research conducted by Hadicka et al. (Ref. 298), a combination of *Lactobacillus* strains such as *Lactobacillus plantarum* VD23, C28 and MS18 and *Lactobacillus* (*Ligilactobacillus*) *salivarius* MS3, MS6 and MS16 demonstrated significant anti-tumour effects while restoring microbial balance in a rat model of CRC. In the investigation by Jacouton et al. (Ref. 299), the findings revealed that *Lactobacillus* (*Lacticaseibacillus*) *casei* BL23 altered the gut microbiome’s composition, fostering the proliferation of beneficial bacteria and diminishing the presence of pathogenic microbes. Furthermore, this probiotic influenced immune cell populations (Tregs/Th17) and cytokine production (IL-6, IL-17, IL-10 and TGF-β) within colonic tissues, highlighting its immunomodulatory capabilities. These outcomes suggest promising avenues for developing new preventive strategies against CRC.

Several limitations temper the interpretation of probiotics in CRC prevention and *H. pylori* management. Several limitations temper the interpretation of probiotics in CRC prevention and *H. pylori* management. The effects of probiotics are highly strain-specific, and results observed with one strain cannot be generalized to others, even within the same species. Additionally, factors such as dosage, formulation and bacterial viability during storage and gastrointestinal transit significantly influence their efficacy (Ref. 300). Furthermore, inter-individual variability in gut microbiome composition can lead to heterogeneous responses to probiotic interventions, limiting their reproducibility and clinical predictability (Ref. 301). Importantly, much of the current evidence is derived from in vitro studies or animal models, with relatively limited large-scale, well-controlled clinical trials in humans. Consequently, while probiotics hold potential as adjunctive strategies, their clinical translation for CRC prevention should be approached with caution and further standardized, strain-specific clinical investigations are required to establish efficacy, optimal dosing and long-term safety.

## Conclusions and future prospects

The association between *H. pylori* infection and CRC highlights the need to better define microbial contributions to colorectal carcinogenesis. This review highlights the multifaceted mechanisms through which *H. pylori* may contribute to CRC risk, including direct effects via secreted toxins, alterations in gastric physiology leading to hypergastrinaemia and indirect pathways mediated by changes in gut microbiota and immune responses. The evidence suggests that *H. pylori* infection may be associated with CRC-related outcomes beyond gastric disease; however, its direct contribution to colorectal carcinogenesis remains to be fully established. However, because much of the current human evidence is observational, causal inferences should be made cautiously in the presence of potential confounding and bias. Given the reported associations between *H. pylori* infection and CRC-related outcomes, future research should prioritize several key areas. First, it is hypothesized that *H. pylori*-derived OMVs serve as long-distance vehicles that transport CagA and VacA specifically to colonic crypt stem cells, triggering early epigenetic shifts before visible dysbiosis occurs. Testing this could clarify whether and how a gastric pathogen may contribute to distal colorectal tumour-promoting processes. Second, we propose a ‘synergistic niche’ hypothesis: the morphological shift to coccoid forms in the colon may alter the local pH and metabolite profile, specifically favouring the growth and establishment of pro-tumorigenic bacteria. Investigating these molecular interactions will move the field from observational associations to mechanistic certainty.

Furthermore, there is a pressing need for longitudinal studies that assess the impact of *H. pylori* eradication on CRC incidence and progression. Such studies could provide valuable insights into whether early intervention can mitigate CRC risk in populations with high prevalence rates of *H. pylori* infection. Additionally, exploring the gut microbiome’s role in modulating the effects of *H. pylori* is essential. As emerging evidence suggests that dysbiosis may influence CRC development, understanding how *H. pylori* interacts with other microbial communities could reveal novel therapeutic targets for prevention and treatment. Finally, public health strategies should consider the implications of these findings by integrating screening for *H. pylori* into CRC prevention programmes, especially in high-risk populations. By addressing both microbial and lifestyle factors associated with CRC, we can develop more effective strategies for reducing its incidence and improving patient outcomes.

In summary, while significant progress has been made in understanding the relationship between *H. pylori* and CRC, much remains to be discovered. Continued interdisciplinary research efforts will be vital in unravelling these complex interactions and translating findings into clinical practice to combat one of the leading causes of cancer mortality worldwide. A deeper understanding of *H. pylori*–host interactions could revolutionize CRC management by enabling precision diagnostics, such as microbial and host genetic profiling to stratify high-risk patients, and targeted therapies disrupting virulence factors like CagA or VacA to halt tumorigenesis. These personalized strategies, integrating microbiome dynamics and host susceptibility, hold transformative potential to reduce CRC burden and improve outcomes in high-prevalence populations.

## Abbreviations

AP: adenomatous polyp
APC: adenomatous polyposis coli
ATG: antithymoglobulin
Bcl-2: B-cell lymphoma-2
CARD10: caspase recruitment domain 10
CagA: cytotoxin-associated gene A
Cag PAI: cag pathogenicity island
CI: confidence interval
COX-2: cyclooxygenase 2
CP: colorectal polyp
CRC: colorectal cancer
CTLA-4: cytotoxic t-lymphocyte-associated protein 4
DC: dendritic cell
ECP: eosinophil cationic protein
EMT: epithelial–mesenchymal transition
ERK: extracellular signal-regulated kinase
FoxP3: Forkhead box protein 3
GC: gastric cancer
HcpC: *Helicobacter* cysteine-rich protein
HP: hyperplastic polyp
IBD: inflammatory bowel disease
ICAM-1: intracellular adhesion molecule 1
IFN-γ: interferon-gamma
IL: interleukin (e.g. IL-1β, IL-6, IL-8, IL-10, IL-17)
IRAK1: interleukin 1 receptor-associated kinase 1
JAK-STAT3: janus kinase signal transducer and activator of transcription 3
LAMP2: lysosome-associated membrane protein 2
LC3: microtubule-associated protein light chain 3
lncRNA: long non-coding RNA
LPS: lipopolysaccharide
MAPK: mitogen-activated protein kinase
miRNA: MicroRNA
MTOR: mechanistic target of rapamycin
MTORC1: mechanistic target of rapamycin complex 1
NADPH: nicotinamide adenine dinucleotide phosphate
NF-κB: nuclear factor kappa-light-chain-enhancer of activated B cells
NO: nitric oxide
NOD1: nucleotide-binding oligomerization domain-containing protein 1
OMP: outer membrane protein
OMV: outer membrane vesicle
OR: odds ratio
PAMP: pathogen-associated molecular pattern
PD-L1: programmed cell death ligand 1
PG: peptidoglycan
PP: Peyer’s patch
PRR: pattern recognition receptor
RAB7: Ras-related protein 7
RECK: Reversion-inducing-cysteine-rich protein with Kazal motifs
ROS: reactive oxygen species
SabA: sialic acid-binding adhesin
SCFA: short-chain fatty acid
SMAD2: Sma- and Mad-related protein 2
SQSTM1: sequestosome protein 1
SSA: sessile serrated adenoma
T4SS: type IV secretion system
TA: tubular adenoma
TGF-β: transforming growth factor beta
TGF-βR2: transforming growth factor Beta receptor 2
TIMP-1: tissue inhibitor of metalloprotease 1
TLR4: Toll-like receptor 4
TNM: tumour, node, metastasis
Treg: regulatory T-cell
TSA: traditional serrated adenoma
TVP: tubulovillous polyp
ULK1: UNC-like autophagy-activating kinase 1
VacA: vacuolating cytotoxin A
VBNC: viable but non-cultivable
vOTU: viral operational taxonomic unit
VP: villous polyp
ZEB1: zinc-finger E-box-binding homeobox 1
ZFAS1: zinc finger antisense 1.

## Long descriptions

### Table 1. Long description

The table is organized into 11 columns: Study design, Outcome category, Sample size (Cases, Control), Crude (O R, Lower, Upper), Adjusted (O R, Lower, Upper), and Reference.

Key data points include:

* Neoplasms (adenoma polyp plus C R C): Four studies listed. A case-control study (Ref. 54) shows an adjusted O R of 1.90 (1.23 to 2.93). A cross-sectional study (Ref. 52) shows a crude O R of 4.07 (2.26 to 7.35).

* Advanced neoplasms: Five studies listed. A cross-sectional study (Ref. 52) shows a high crude O R of 9.75 (4.31 to 21.20).

* C R C: Twenty-four studies listed. A large retrospective study (Ref. 58) with 82,420 cases and over 47 million controls shows an adjusted O R of 1.89 (1.69 to 2.10). A case-control study (Ref. 61) shows a high adjusted O R of 10.60 (2.70 to 41.30).

* Overall colorectal polyps: Eight studies listed. A retrospective study (Ref. 81) shows a crude O R of 5.23 (3.60 to 7.70).

* Adenoma polyps: Seventeen studies listed. A cross-sectional study (Ref. 87) shows an adjusted O R of 3.24 (2.18 to 4.80).

* Advanced adenoma polyps: Five studies listed. A case-control study (Ref. 67) shows a crude O R of 1.80 (1.69 to 1.92).

* Hyperplastic polyp: Four studies listed. A retrospective cohort (Ref. 94) shows an adjusted O R of 1.23 (1.18 to 1.28).

* Single vs. Multiple polyps: Multiple polyps generally show higher O R values, such as Ref. 68 showing an adjusted O R of 1.98 for single polyps versus 2.41 for two or more polyps.

* Polyp size: Ref. 68 shows an adjusted O R of 2.33 for polyps greater than or equal to 10 mm and 2.15 for polyps less than 10 mm.

Navigate back to Table 1..

### Table 2. Long description

The table is organized into eight columns: Study design, Study model, Sample size, Methodology, Statistical methods used, Microbiota changes, Potential functional implications, and References.

Key findings include:

* Animal models (Mongolian gerbils and mice) show increases in E. coli, Enterococcus, Akkermansia, and Ruminococcus, linked to intestinal inflammation and tumor progression.

* Human studies (children and adults) frequently report increases in Prevotella, Proteobacteria, and Firmicutes, while often showing decreases in Subdoligranulum and Bacteroidetes.

* Methodologies primarily involve 16S r R N A sequencing, P C R, and meta-genomic sequencing.

* Functional implications across studies highlight gut homeostasis disruption, promotion of systemic low-grade inflammation, impaired metabolic function, and potential mucosal barrier impairment.

* Statistical methods include Mann-Whitney U tests, Student's t tests, A N O V A, and P E R M A N O V A.

* Sample sizes range from small groups of 17 participants to larger cohorts of 428 participants.

Navigate back to Table 2..

### Figure 1. Long description

A flowchart titled H. pylori Infection at the top center.

* The left side, labeled Direct mechanisms in blue, leads to a box containing Virulence factors and Hypergastrinemia. A blue arrow continues from this box down to the final outcome, C R C.

* The right side, labeled Indirect mechanisms in orange, leads to a box containing Morphological change, Intracellular lifestyle, Extracellular vesicle release, Autophagic process, H. pylori–n c R N A interaction, and Gut microbiota changes. An orange arrow continues from this box down to the final outcome, C R C.

* A central red pathway connects both side boxes to a vertical series of red ovals. The sequence is as follows:

1. Inflammation.

2. Chronic infection and Immune response disruption (side-by-side ovals).

3. Pro-inflammatory intestinal environment.

4. Tumor-promoting microenvironment.

All pathways converge at the bottom box labeled C R C.

Navigate back to Figure 1..

### Figure 2. Long description

The diagram is divided into three horizontal sections.

Top Section: A human silhouette shows the digestive tract. Arrows indicate a decrease in gastric acid and an increase in Gastrin. To the right, H. pylori bacteria interact with the intestinal epithelium, releasing O M V s and causing microbiota dysbiosis. A legend identifies components like the Type 4 secretion system, vac A, cag A, L P S, and various immune cells.

Middle Section: A zoomed-in view of the epithelial membrane. On the left, a purple box lists Inflammation, E M T, and Tumor growth, influenced by m i R N A s like m i R-21 and m i R-146 a. In the center, the Type 4 secretion system and other receptors trigger pathways leading to R O S production and D N A damage. On the right, a T naive cell differentiates into T H 1, T H 17, or T reg cells, releasing cytokines like I F N gamma, I L-17, and I L-10.

Bottom Section: Two distinct panels. The left panel (blue background) details the Autophagy process from Initiation to Autophagosome formation, involving P I 3 K, m T O R, and A T G complexes. This process leads to H. pylori survival and cancer progression. The right panel (yellow background) summarizes the immune effects: Inflammation, Promotes tumor immunity, and Immune suppression.

Navigate back to Figure 2..

### Figure 3. Long description

The diagram is divided into two main sections.

Top section:

At the top, H. pylori bacteria and red dots labeled Virulence factor are in the gastric lumen. An arrow shows a virulence factor passing between pink columnar epithelial cells to reach a D C (dendritic cell) below. The D C points to a row of blue T naive cells. From these, arrows branch into C D 4 (green) and C D 8 (light blue) cells. The C D 4 cell further differentiates into three types: Th 1 (purple), Th 17 (brown), and F O X P 3 T reg (orange). Each of these three cells releases signaling molecules represented by small colored dots. A flat-headed inhibitory line extends from the F O X P 3 T reg cell toward the C D 8 cell.

Bottom section:

A rectangular box titled C D 4 and C D 8 T cell dynamics is split diagonally. The left side, shaded green and labeled Acute phase, shows a high density of light blue C D 8 cells and fewer green C D 4 cells. The right side, shaded pink and labeled Chronic phase, shows a shift to a high density of green C D 4 cells and fewer light blue C D 8 cells.

Navigate back to Figure 3..

### Figure 4. Long description

The diagram is divided into two main sections.

On the left, an anatomical cross-section of a gastric crypt shows the transition from a healthy state to DYSBIOSIS, indicated by a green-to-red arrow at the bottom. The crypt is lined with epithelial cells and contains a central area labeled Gut microbiota. At the top left, H. pylori spiral forms, coccoid forms, and O M V s are shown. A group of PROBIOTICS is shown entering the crypt to interact with these pathogens and the resident microbiota.

On the right, a flowchart titled Possible mechanisms lists specific probiotic strains and their actions:

* Lactobacillus gasseri O L L 2 7 1 6 leads to Conversion of H. pylori, which Prevents coccoid proliferation.

* Lactococcus lactis subsp. lactis A 1 6 4 produces Lacticin, which Reduces H. pylori population and Damages coccoid surface.

* Lactobacillus reuteri produces Reuterin, which Inhibits H. pylori colonization and adhesion.

* Lactobacillus gasseri A T C C 3 3 3 2 3 Modulates H. pylori-induced autophagy.

* Limosilactobacillus fermentum G R-3 produces S C F A, which, along with Lactobacillus plantarum, Lactobacillus salivarius, and Lactobacillus casei, leads to Restoration of gut microbiota balance and Anti-tumor effects.

* Lactobacillus rhamnosus and Escherichia coli Nissle 1 9 1 7 act on tight junction proteins J A M, Occludin, and Claudin to Enhances intestinal integrity.

Navigate back to Figure 4..
